# A Randomized Placebo-Controlled Phase 2 Study of Gemcitabine and Capecitabine with or without T-ChOS as Adjuvant Therapy in Patients with Resected Pancreatic Cancer (CHIPAC)

**DOI:** 10.3390/pharmaceutics14030509

**Published:** 2022-02-25

**Authors:** Susann Theile, Julia Sidenius Johansen, Dorte Lisbet Nielsen, Benny Vittrup Jensen, Carsten Palnæs Hansen, Jane Preuss Hasselby, Sverrir Vídalín Eiríksson, Inna Markovna Chen

**Affiliations:** 1 Department of Oncology, Herlev and Gentofte Hospital, Copenhagen University Hospital, 2730 Herlev, Denmark; julia.sidenius.johansen@regionh.dk (J.S.J.); dorte.nielsen.01@regionh.dk (D.L.N.); benny.vittrup.jensen@regionh.dk (B.V.J.); inna.chen@regionh.dk (I.M.C.); 2 Department of Medicine, Herlev and Gentofte Hospital, Copenhagen University Hospital, 2730 Herlev, Denmark; 3 Department of Clinical Medicine, Faculty of Health and Medical Sciences, University of Copenhagen, 2200 Copenhagen, Denmark; 4 Department of Surgery, Rigshospitalet, Copenhagen University Hospital, 2100 Copenhagen, Denmark; carsten.palnaes.hansen@regionh.dk; 5 Department of Pathology, Rigshospitalet, Copenhagen University Hospital, 2100 Copenhagen, Denmark; jane.preuss.hasselby@regionh.dk; 6 Genís hf., 580 Siglufjörður, Iceland; sverrir.vidalin.eiriksson@genis.is

**Keywords:** adjuvant chemotherapy, chitinase 3-like 1 protein, chitooligosaccharide, pancreatic cancer, YKL-40

## Abstract

The antitumor activity of chitooligosaccharides has been suggested. This phase 2 trial evaluated the efficacy and safety of T-ChOS™, in addition to adjuvant chemotherapy, in patients after resection of pancreatic ductal adenocarcinoma (PDAC). In this single-center, randomized, double-blind, placebo-controlled trial using patients ≥18 years of age after complete macroscopic resection for PDAC, patients were randomly assigned (1:1) to either a continuous oral T-ChOS group or a placebo group, in combination with gemcitabine (GEM) and oral capecitabine (CAP), for a maximum of six cycles. The primary endpoint was disease-free survival (DFS). Recruitment was stopped prematurely in July 2018, with 21 of planned 180 patients included, due to poor accrual and because modified FOLFIRINOX replaced GEM/CAP for the target population. Nine patients received T-ChOS and twelve received the placebo. The median DFS was 10.8 months (95% CI 5.9–15.7) for the T-ChOS arm and 8.4 months (95% CI 0–21.5) in the placebo arm. Overall, seven patients (78%) in the T-ChOS arm and eight patients (67%) in the placebo arm experienced at least one grade 3–4 treatment-related adverse event, most frequently neutropenia. Altogether, the addition of T-ChOS to chemotherapy in patients after resection of PDAC seems safe. However, the clinical benefit cannot be assessed due to the premature cessation of the trial.

## 1. Introduction

Pancreatic cancer is one of the leading causes of cancer-related death in Western countries, with an estimated number of deaths of 92,100 in Europe [1] and 47,050 in the United States, in 2020 [2]. Resection is the only potentially curative treatment; however, only 20% of patients present with resectable disease [3]. For patients that undergo resection of the tumor followed by adjuvant chemotherapy, 5-year survival rates up to 29% have been reported in randomized trials over the past two decades [4,5,6].

Chitooligosaccharides (COS) are oligomers that are depolymerized from chitosan, a natural nontoxic biopolymer produced by the deacetylation of chitin, which is a major component of the shells of crustaceans. COS have been reported to possess a range of beneficial biological effects, including antimicrobial, antioxidant, anti-inflammatory, immunostimulatory, and the inhibition of tumor proliferation [7,8,9]. COS suppress the growth of various cancer cells in vitro, such as prostate, lung, hepatocellular, and colon cancer [10,11,12]. In vivo, COS have been shown to inhibit tumor growth of HepG2 xenografts, and in a Lewis lung carcinoma (LLC)-bearing mouse model, COS inhibited tumor growth as well as the development of lung metastasis [13]. In addition, COS have been reported to inhibit tumor growth of colorectal cancer cells in mice models in vivo [14]. Thus, treatment with COS has been the topic of several preclinical studies in the cancer field because of their ability to reduce chronic inflammation, inhibit tumor angiogenesis, and stimulate the immune system. In general, COS are expected to be safe for humans to consume.

Tumor-promoting inflammation, one of the conceptual hallmarks of cancer development and progression [15], has a major role in the development and progression of pancreatic ductal adenocarcinoma (PDAC) [16,17]. Many proteins (e.g., Interleukin-6 (IL-6) and YKL-40/chitinase 3-like 1 protein (CHI3L1)) secreted by cancer cells, macrophages, neutrophils, and fibroblasts stimulate inflammation. IL-6 is a pleiotropic cytokine that plays a major role in angiogenesis, cancer cell survival, chemotherapy resistance, and the development of liver metastases [18,19,20,21,22]. YKL-40 regulates the vascular endothelial growth factor and promotes angiogenesis, protects against apoptosis, and stimulates tumor progression and metastasis [23]. Moreover, it has also been demonstrated that YKL-40 is a highly conserved heparin-, chitin-, and collagen-binding glycoprotein belonging to the glycosyl hydrolase family 18, although it lacks chitinase/hydrolase activity [23]. Several studies also demonstrated that high circulating IL-6 and YKL-40 levels in patients with different types of cancer, including PDAC, are associated with poor prognosis [24,25]. Recently, a role of CHI3L1/YKL-40 in resistance to gemcitabine (GEM) in PDAC treatment has been suggested [26].

In this study, we used T-ChOS™, an oral nutritional supplement that is an optimized mixture of heterooligosaccharides derived from chitin, and composed of two monomeric moieties, glucosamine and N-acetyl glucosamine. T-ChOS chitooligosaccharides have been selected due to their high bioactivity in inflammatory models and strong binding affinity to YKL-40 [27]. The binding of T-ChOS to YKL-40 involves the heparin domain of YKL-40 [27].

We aimed to investigate GEM and capecitabine (CAP) with or without T-ChOS as adjuvant therapy in patients with resected PDAC, with disease-free survival (DFS) as the primary endpoint. The study was prematurely terminated due to slow recruitment and strong evidence that the combination chemotherapy with fluorouracil, folinic acid, irinotecan, and oxaliplatin (modified FOLFIRINOX, with no bolus fluorouracil and 150–180 mg/m^2^ dose of irinotecan) is a more effective adjuvant therapy in patients after resection of PDAC compared to GEM-based therapy [28]. We present the results of the final analysis for the trial.

## 2. Materials and Methods

### 2.1. Study Population

Eligible patients were ≥18 years of age with histologically confirmed PDAC after macroscopic complete resection (R0 and R1, according to National Danish guidelines [29]). A maximum of 12 weeks between resection and treatment initiation was allowed. Additional criteria were the Eastern Cooperative Oncology Group (ECOG) performance status (PS) 0–1 as well as the hematology and serum biochemistry (including creatinine, bilirubin, and liver enzymes) values appropriate to receive chemotherapy. Presence of metastatic or locally recurrent PDAC was an exclusion criterion. Prior neoadjuvant treatment or other systemic or radiation therapy for PDAC were not allowed. Patients with prior malignancy were allowed, provided it was adequately treated basal carcinoma or squamous cell carcinoma of the skin, or in situ cervix carcinoma or incidental prostate cancer (T1a, Gleason score ≤ 6, PSA < 0.5 ng/mL), or patients had a DFS of >5 years from any other malignancy. Other exclusion criteria were: patients with a history of serious or concurrent illness or uncontrolled medical disorder, any medical condition that might be aggravated by chemotherapy or that could not be controlled, known or suspected allergy to the investigational agents, patients receiving other investigational agents, inability to comply with study procedures/schedule, and pregnant or nursing women. In addition, subjects that required the use of anticoagulation therapy (unfractionated or low molecular weighted heparin) were also considered ineligible.

### 2.2. Study Design and Objectives

The study was a single-center, randomized, double-blind, placebo-controlled phase 2 trial to investigate the efficacy and safety of the nutritional supplement T-ChOS in combination with adjuvant chemotherapy GEM/CAP in patients with surgically resected PDAC, conducted at the Department of Oncology, Herlev and Gentofte Hospital, Herlev Denmark. Patients were randomized 1:1 using permutated block randomization with stratification based on the nodal status (lymph node negative versus lymph node positive) and resection status (R0—tumor-free margin versus R1—microscopically positive margin).

The trial was prematurely closed for recruitment in July 2018, with 21 out of planned 180 patients included, due to poor accrual and because modified FOLFIRINOX replaced GEM/CAP for the target population. Ongoing patients were unblinded, and apart from intake of placebo, treatment, including chemotherapy, if applicable, and assessments were continued as per the protocol.

The objective of the trial was to compare the efficacy, safety, and quality of life (QoL) of patients receiving GEM/CAP/T-ChOS versus GEM/CAP/Placebo. The primary endpoint was DFS. Secondary endpoints were overall survival (OS), safety, and tolerability of the combinational treatment and QoL.

This study followed the Consolidated Standards of Reporting Trials (CONSORT) reporting guidelines (Supplemental Table S1).

### 2.3. Study Treatment

In a blinded fashion, patients were to receive capsules of T-ChOS, or matched placebo, 600 mg daily for continuous oral intake. T-ChOS and placebo pharmaceuticals were provided by Genís hf, Siglufjörður, Iceland, and both were encapsulated at the Central Pharmacy for Capital Region of Denmark, Herlev. Upon confirmation of eligibility by the investigator, central randomization was performed at the pharmacy, Herlev Hospital, and patient-specific, blind-labelled study drugs were dispensed to the patients. Blinding was maintained for patients and investigators until the premature end of recruitment. Backbone chemotherapy consisted of intravenous administration of GEM 1000 mg/m^2^ on day 1, day 8, and day 15 of every 28-day cycle, and oral intake of tablet CAP 830 mg/m^2^ twice daily for 21 days out of the 28 days of a treatment cycle. Patients received GEM/CAP for a planned total of 6 months/cycles, followed by continuation of the oral T-ChOS/placebo. Patients could continue treatment with T-ChOS/placebo for a maximum of 5 years, or until disease recurrence, death, unacceptable toxicity, or withdrawal of consent. Chemotherapy was to be modified (delay/interruption/dose reduction) in case of significant hematological and/or non-hematological toxicity, according to institutional standards. Dose reduction of T-ChOS/placebo was not allowed. T-ChOS/placebo was to be discontinued in case of thromboembolic event or requirement to start treatment with heparin.

### 2.4. Study Assessments and Procedures

Complete medical history and physical examination were required at baseline, as well as electrocardiogram and appropriate laboratory tests to assess blood count; clinical biochemistry, including serum CA 19-9; and C-reactive protein (CRP) levels. Evaluations at baseline also included postoperative CT and/or MRI scan and PS assessment. Prior to the start of a new cycle, the status of the patient was assessed by means of physical examination, PS, complete blood counts, and blood biochemical testing. Blood counts and adverse events were assessed prior to each treatment. CT and/or MRI scans and measurements of serum CA 19-9 levels were repeated every 3 months in the first year, every 4 months in the second and third year, and every 6 months in successive years up to five years, and/or until disease progression. Adverse events were graded according to the National Cancer Institute Common Terminology Criteria for Adverse Events, version 4.0. After disease recurrence, patients were followed for OS via electronical records.

### 2.5. Translational Research

Patients participating in the trial were asked if they would participate concomitantly in the BIOPAC study (NCT03311776), a national biomarker study for patients with pancreatic cancer. For patients that consented to participate in BIOPAC, additional IL-6 and YKL-40 analyses were performed prospectively and blinded to clinical data. Pretreatment and planned longitudinal (before the second cycle, and at the timepoint of each CT evaluation) serum concentrations of IL-6 were determined in duplicate by an enzyme-linked immuno-sorbent assay (ELISA) (Human IL-6 Quantikine HS ELISA kit, Cat. #HS600, R&D Systems, Abingdon, Oxon, UK) according to the manufacturers’ instructions. The detection limit of the IL-6 ELISA is 0.01 ng/L and intra- and inter-assay coefficients of variations (CVs) are ≤8% and ≤11%. The median serum IL-6 level in healthy subjects is 1.4 ng/L (5–95% reference levels; 0.51–4.92 ng/L) [30]. Pretreatment and planned longitudinal (before the second cycle, and at the timepoint of each CT evaluation) serum concentrations of YKL-40 were determined by a commercial ELISA (MicroVue YKL-40 EIA, Cat. #8040, Quidel, CA, USA). The detection limit of the YKL-40 ELISA is 10 μg/L. The intra-assay and inter-assay CVs are <5% and <6%, respectively [31]. Serum CA19-9 was determined using the Immulite 2000 GI-MA assay (Cat. #L2KG12, Siemens), which is a solid-phase, two-site sequential chemiluminescent immunometric assay. Elevated serum CA19-9 is defined as >37 × 10^3^ IU/L. Serum C-reactive protein (CRP) was analysed using a Sentinel CRP Ultra ready-to-use, liquid assay reagent using an immunoturbidimetric method on a fully automated chemistry analyser (Kit-test SENTINEL CRP Ultra (UD), 11508 UD-2.0/02 2015/09/23, SENTINEL CH. SpA, Milano, Italy). The measurement range for CRP is 0.3–640 mg/L with an intra-assay CV of 3%, and an inter-assay CV of <15%. Elevated serum CRP is defined as >10 mg/L.

### 2.6. QoL

QoL was assessed using the European Organization for Research and Treatment of Cancer quality of life questionnaire (EORTC QLQ) C-30, version 3 (EORTC Quality of Life Group, Brussels, Belgium) [32,33]. Patients were asked to fill in the EORTC QLQ-C30 at baseline, and every 3 months in the first year, every 4 months in the second and third year, and every 6 months in successive years up to five years. Scores were derived and scaled from 0 to 100 according to the EORTC scoring manual [34]. Descriptive statistics were employed to obtain mean values at the different timepoints. Baseline assessment was compared between groups using t-tests.

### 2.7. Statistical Methods

Based on the assumption that the median DFS after adjuvant treatment with conventional treatment was approximately 14 months, and a true hazard ratio (HR) for the control arm relative to the experimental arm of 0.6, we needed to study 90 patients in each treatment arm to be able to reject the null hypothesis that the experimental and control survival curves were equal, with a power of 90% and a 10% significance level.

The primary endpoint was DFS, measured as the time from randomization to the date of locoregional tumor recurrence and/or distant metastases, or death from any cause. Patients without event at the point of final analysis were censored at the date of last CT scan. Secondary endpoint was OS, measured as the time from randomization until death from any cause. Patients still alive at the point of final analysis were censored at the last date known to be alive. Median survival times were estimated using the Kaplan–Meier method, and differences in survival times were tested using the log-rank test. Proportional hazards regression was used to estimate the effect of treatment on DFS. Descriptive methods were used for patient characteristics, exposure to treatment, adverse events, and QoL. All statistical analyses were performed with SPSS version 25. A two-sided *p*-value < 0.05 was considered significant.

## 3. Results

### 3.1. Patient Inclusion

**Figure 1:**
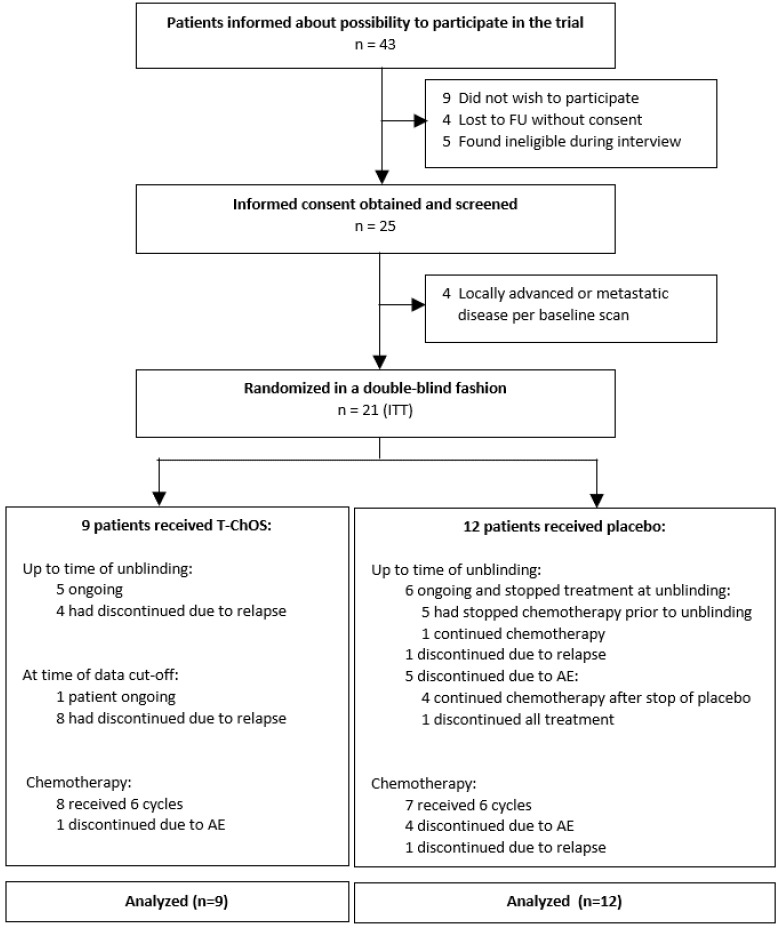
Study CONSORT flow diagram. AE, adverse event; FU, follow-up; ITT, intent-to-treat.

The trial was opened for recruitment in December 2016 and prematurely closed in July 2018 due to low accrual rate and because modified FOLFIRINOX replaced the GEM/CAP combination as the standard adjuvant chemotherapy for fit patients after surgical resection of PDAC, such as the target population for this trial. During that period, 43 potential patients were recruited. At the time the recruitment was stopped, 21 of the planned 180 patients had been enrolled and randomized to receive treatment. Additionally, four patients consented to participate and were screened, however, they were not randomized due to ineligibility, i.e., baseline scan showed locally advanced or metastatic disease (Figure 1).

At the time of recruitment termination, 13 of the ongoing 15 patients were still receiving treatment. The ongoing patients were informed about the possibility of unblind treatment allocation, and all patients consented. Patients that received treatment with T-ChOS (*n* = 5) could continue treatment as planned per protocol. Patients in both treatment arms continued to receive backbone chemotherapy with GEM/CAP for the planned six cycles if applicable (*n* = 2). The disease assessment was continued for all patients as per protocol. The final unmasking at the time of analyses revealed that 12 patients had received the placebo and 9 patients had received treatment with T-ChOS. The demographics and disease characteristics in both arms are comparable as shown in Table 1.

### 3.2. Treatment

At the time of unblinding, the median actual exposure for patients receiving T-ChOS was 276 days (range 73–399 days). For patients receiving the placebo, the median actual exposure was 143 days (range 11–558) (Table 2). For all patients in the T-ChOS arm that stopped treatment (*n* = 8), the discontinuation was due to relapse of disease. In the placebo arm, one patient had stopped treatment due to relapse of disease, six patients stopped treatment at the time of unblinding, and five patients had discontinued treatment due to adverse events, i.e., thromboembolic event that required treatment with heparin (*n* = 3), or generalized rash (*n* = 2). The median number of cycles of GEM was six (range 1–6) in both arms, with a median relative dose intensity of 89% in the T-ChOS arm and 86% in the placebo arm. CAP was given for a median of six cycles (range 1–6) in the T-ChOS arm and for a median of four cycles (range 1–6) in the placebo arm. The median relative dose intensity for CAP was 65% in the T-ChOS arm and 73% in the placebo arm (Table 2). The permanent discontinuation of all chemotherapy due to adverse events was necessary in 1 out of 9 patients in the T-ChOS arm, and in 4 out 12 patients in the placebo arm.

### 3.3. Efficacy

**Figure 2:**
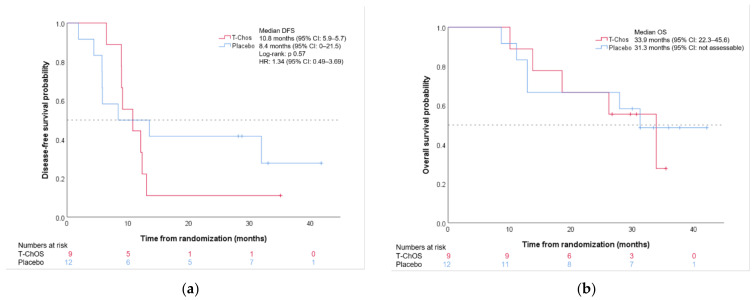
Kaplan–Meier plot for disease-free survival (**a**) and overall survival (**b**).

At the time of data cut-off (July 2020), one patient in the T-ChOS arm and four patients in the placebo arm were recurrence-free. The median DFS was 10.8 months (95% CI 5.9–15.7) for the T-ChOS arm and 8.4 months (95% CI 0–21.5) in the placebo arm (log-rank *p* = 0.57, HR 1.34, 95% CI 0.49–3.69) (Figure 2a). The median OS was 33.9 months (95% CI 22.3–45.6) in the T-ChOS arm and 31.3 months (95% CI not assessable) in the placebo arm (Figure 2b).

### 3.4. Safety

The safety profile of the study treatment was similar in both arms. The treatment-related adverse events (Table 3) were characterized by the known side effects from the background adjuvant chemotherapy combination of GEM/CAP. The most common hematological toxicity was neutropenia, with grade 3–4 events for 44% of patients in the T-ChOS arm and 58% of patients in the placebo arm. Fatigue and hand-foot syndrome was reported for at least two thirds of patients in both arms were the most common non-hematological toxicities. In addition, gastrointestinal toxicities, i.e., diarrhea, nausea, anorexia, and mucositis, were commonly reported for approximately 50% of patients in both arms. Infections were reported for 44% in the T-ChOS arm and 42% in the placebo arm. No treatment-related deaths were observed. One female patient in the placebo arm developed breast cancer approximately 18 months after the randomization in the trial. The event was considered unrelated to the study treatment.

### 3.5. Translational Research

Concomitantly, for translational research, we aimed to collect blood samples for longitudinal analyses of serum YKL-40 and IL-6, additional to the routinely determined serum CRP and CA19-9 concentrations. For logistic reasons, we failed to collect the samples continuously as planned. The median values for serum concentrations of YKL-40, IL-6, CRP, and CA19-9, together with the number of contributing samples, are presented in Supplemental Table S2 for the assessment during the first 6 months. The distribution of the particular values at the different timepoints during the first 6 months are displayed in the graphs in Supplemental Figure S1. Due to the low number of patients, further formal analyses were not performed.

### 3.6. Quality of Life

The compliance regarding the return of the QoL questionnaire was unfortunately very low, especially for the post-baseline evaluations. Whereas at baseline, EORTC QLQ-C30 was answered by 9/9 patients in the experimental T-ChOS arm and 10/12 patients in the placebo arm, a maximum of respectively 3 and 6 answered questionnaires were retrieved at any of the post-baseline timepoints. Supplemental Table S3 presents simple descriptive analyses of the global health status, and the five functional scales (symptom scales omitted) at the timepoints up to 1 year after treatment started; however, a paired analysis to compare timepoints was not performed due to the high proportion of missing assessments within the small sample size. A t-test revealed a significant difference (*p* = 0.024) in the self-assessed global health status, with mean values at 70.4 and 55.0 for patients in the T-ChOS arm and in the placebo arm, respectively.

## 4. Discussion

This trial aimed to evaluate the antitumor activity of chitooligosaccharides, in combination with chemotherapy, in a randomized clinical trial. At the time of designing the trial, the combination of GEM/CAP was chosen as backbone therapy for the adjuvant treatment of patients after surgical resection of PDAC, based on the first results presented for the ESPAC-4 trial [35]. However, we decided to close the recruitment period for the trial prematurely, in July 2018, due to low recruitment, and after the presentation of data showing that modified FOLFIRINOX was significantly more effective than GEM in combination with CAP as adjuvant chemotherapy for fit patients after surgical resection of PDAC, such as the target population for this trial [36]; modified FOLFIRINOX had subsequently been implemented as standard adjuvant treatment at our site as well. Thus, this single-center trial included 21 patients out of the planned 180 patients. For ethical reasons, we informed and unblinded the ongoing patients. Patients that had received treatment with T-ChOS continued treatment as per protocol, including the planned chemotherapy with GEM/CAP. For patients in the placebo arm, the ongoing chemotherapy was continued as per protocol; however, the intake of placebo capsules was discontinued. The assessment for the primary endpoint, DFS, was continued for all patients as planned, though the blinding of treatment allocation was voided.

Limited conclusions from this prematurely terminated trial can be drawn. The combination of chemotherapy with oral nutritional supplement COS, such as T-ChOS, seems feasible without additional toxicities. Noteworthy, five out of nine patients underwent T-ChOS treatment for more than a year, without reporting side effects, after completion of the backbone chemotherapy, underlining the safety of long-term exposure to COS as a nutritional supplement. The observed median OS of 31.3 months (95% CI 23.3–39.3) overall for the intent-to-treat population is in line with the 28 months reported for the GEM + CAP arm in the ESPAC-4 trial [6]. Bearing in mind the low number of patients, it remains that the median DFS seen in this trial is shorter than the 13.9 months observed for GEM + CAP in the ESPAC-4 trial [6], or for that matter, those of approximately 13–14 months observed for gemcitabine alone in both the ESPAC-4 and other trials [6,28,37]. This might partly reflect the necessity of dose reduction, or even the premature discontinuation of the chemotherapy, due to the relatively high proportion of patients with hand-foot syndrome, as well as grad 3–4 neutropenia, in both arms of our trial. Moreover, we had no exclusion criteria concerning the baseline/postoperative serum CA19-9 level as, for example, in CONKO-001 [37]. Thus, four patients with baseline serum CA19-9 of >2.5 × ULN were included, and these were all randomized to the placebo arm. Postoperative serum CA19-9 has been shown to be an independent predictor for OS [6,38]. The interpretation of our findings is limited given the substantially reduced power for formal statistical testing. Hence, the suggested antitumor or chemopreventive activity of COS could not be investigated properly. The preclinical research, most recently reviewed by Zhai et al., suggests a range of applications for COS in cancer treatment; however, clinical trials are missing [9]. Considering the favorable safety profile of COS, clinical trials conducted to investigate their antitumor activity are warranted. At present, T-ChOS, or its successor product SimeCOS, are mostly used as nutritional supplements, because of their anti-inflammatory activities. At our site, we currently have another double-blind, randomized trial (VEK no. H-18036500) ongoing to investigate whether SimeCOS can reduce musculoskeletal side effects, such as arthralgia and myalgia, in breast cancer patients receiving adjuvant aromatase inhibitor therapy after surgery. Furthermore, the simultaneous targeting of CHI3L1 and the programmed cell death of protein-1/programmed death ligand-1 axis promoted cytotoxicity of CD8+ T cells and tumor cell death [39]. Thus, a combined COS and checkpoint blockade could represent a potential anticancer approach.

We acknowledge that adjuvant therapy for patients surgically resected for PDAC, and recruiting these patients for clinical trials, remains challenging. Up to half of the patients undergoing resection for PDAC did not receive adjuvant therapy for various reasons, including postoperative clinical conditions, poor PS, and early disease progression [40,41,42]. Often, patients with PDAC have comorbidities, and the time from operation to the start of adjuvant treatment is frequently prolonged due to the extensive nature of the surgery, which is associated with a longer recovery time and the incidence of postoperative complications. Additionally, the completion of planned adjuvant chemotherapy is often compromised in patients with PDAC [43]. In the current trial, only 1 of 21 patients could complete the planned six cycles of the adjuvant chemotherapy combination at the intended full dose; whereas the remaining patients required dose reductions of either one or both drugs (GEM/CAP) during early treatment. Valle et al. found, based on data from the ESPAC-3 trial, that timing of adjuvant chemotherapy played less important role and thus possible delay for up to 12 weeks after operation was of no disadvantage in terms of survivals [44]. More important, the same group and others showed that completion of the full course of the planned adjuvant chemotherapy is an independent favorable prognostic factor [44,45]. Compared to the GEM/CAP group in the ESPAC-4 trial, that reported a median dose intensity of 83% for GEM and 78% for CAP [6], the median relative dose intensity in this trial was similar for GEM, 89% for the combined groups, whereas the median relative dose intensity for CAP was considerably lower, 70% for the combined groups.

In the original protocol, we intended to additionally investigate the QoL, and whether serum YKL-40 and IL-6 levels are associated with clinical outcome. We recognize that the restricted number of patients, and lack of longitudinal assessments, for proper paired analyses make drawing conclusions difficult for this part of the trial, and any statement should be made with caution. We found that serum YKL-40 concentrations showed a tendency to increase when patients received T-ChOS, in line with the assumption that YKL-40 is overexpressed to compensate for molecules bound by the experimental product (Supplementary Figure S1a). With regards to QoL, in general, an increase in self-assessed global health status, and almost all the functional scales, were noted in both arms during adjuvant treatment.

## 5. Conclusions

In conclusion, this study demonstrated that T-ChOS seems to be safe to use in conjunction with adjuvant chemotherapy. The clinical efficacy endpoints of DFS and OS are inconclusive due to the premature cessation of the trial.

## Figures and Tables

**Table 1:** Patient characteristics.

		T-ChOS *n* = 9	Placebo *n* = 12	All Included Patients *n* = 21
Characteristics		Number of Patients (%)	Number of Patients (%)	Number of Patients (%)
Median age, years (range)		68 (61–72)	71 (59–80)	71 (59–80)
Gender				
	Male	3 (33.3)	6 (50.0)	9 (42.9)
	Female	6 (66.7)	6 (50.0)	12 (57.1)
ECOG Performance status				
	0	6 (66.7)	10 (83.3)	16 (76.2)
	1	3 (33.3)	2 (16.7)	5 (23.8)
Resection status				
	R0	7 (77.8)	9 (75.0)	16 (76.2)
	R1	2 (22.2)	3 (25.0)	5 (23.8)
Nodal status				
	Lymph node negative	1 (11.1)	3 (25.0)	4 (19.0)
	Lymph node positive	8 (88.9)	9 (75.0)	17 (81.0)
Tumor stage AJCC/UICC 8th edition				
	IB	1 (11.1)	1 (8.3)	2 (9.5)
	IIA	0	2 (16.7)	2 (9.5)
	IIB	5 (55.6)	5 (41.7)	10 (47.6)
	III	3 (33.3)	4 (33.3)	7 (33.3)
Localization of primary tumor				
	Head	8 (88.9)	8 (66.7)	16 (76.2)
	Other	1 (11.1)	4 (33.3)	5 (23.8)
Type of resection				
	Whipple resection	7 (77.8)	6 (50.0)	13 (61.9)
	Distal pancreatectomy	1 (1.11)	4 (33.3)	5 (23.8)
	Total pancreatectomy	1 (11.1)	2 (16.7)	3 (14.3)
Tumor grade				
	Well differentiated	0	1 (8.3)	1 (4.8)
	Moderately differentiated	5 (55.6)	6 (50.0)	9 (42.9)
	Poorly differentiated	3 (33.3)	3 (25.0)	6 (28.6)
	Unknown	1 (11.1)	2 (16.7)	3 (14.3)
Local invasion				
	No	0	2 (16.7)	2 (9.5)
	Yes	7 (77.8)	9 (75.0)	16 (76.2)
	Unknown	2 (22.2)	1 (8.3)	3 (14.3)
Perineural invasion				
	No	3 (33.3)	1 (8.3)	4 (19.0)
	Yes	6 (66.7)	10 (83.3)	16 (76.2)
	Unknown	0	1 (8.3)	1 (4.8)
Postoperative complication				
	No	5 (55.6)	8 (66.7)	13 (61.9)
	Yes	3 (33.3)	4 (33.3)	7 (33.3)
	Unknown	1 (11.1)	0	1 (4.8)
Time from surgery to randomization				
	Median days (range)	42 (29–50)	42 (28–87)	42 (28–87)
Baseline CA19-9				
	Median 10^3^ IU/L (range)	20 (3–73)	44 (4–1430)	26 (3–1430)

**Table 2:** Treatment exposure.

	T-ChOS *n* = 9	Placebo *n* = 12	All Included Patients *n* = 21
**Gemcitabine**			
Median cycles received (range)	6 (1–6)	6 (1–6)	6 (1–6)
Patients receiving 6 cycles, *n* (%)	7 (77.8)	7 (58.3)	14 (66.7)
Median RDI % (range)	88.9 (33.3–100)	86.1 (38.9–100)	88.9 (33.3–100)
Patients with RDI >80%, *n* (%)	7 (77.8)	7 (58.3)	14 (66.7)
**Capecitabine**			
Median cycles received (range)	6 (1–6)	4 (1–6)	5 (1–6)
Patients receiving 6 cycles, *n* (%)	5 (55.6)	5 (41.7)	10 (47.6)
Median RDI % (range)	65.3 (4.8–100)	73.1 (31.7–100)	69.7 (4.8–100)
Patients with RDI >80%, *n* (%)	3 (33.3)	4 (33.3)	7 (33.3)
**T-ChOS™/Placebo**			
**At time of unblinding**			
Median total exposure in days (range)	279 (73–402)	143 (11–589)	212 (11–589)
Median actual exposure in days (range)	276 (73–399)	143 (11–558)	212 (11–558)
**At time of data cut-off**			
Median total exposure in days (range)	374 (212–1078)	NA	284 (11–1078)
Median actual exposure in days (range)	374 (212–1078)	NA	279 (11–1078)

RDI, relative dose intensity; Total exposure = last day of treatment − first day of treatment + 1; Actual exposure = last day of treatment − first day of treatment + 1 − days with treatment interruption.

**Table 3:** Treatment-related adverse events.

	Gemcitabine + Capecitabine + T-ChOS *n* = 9		Gemcitabine + Capecitabine + Placebo *n* = 12	
	All Grades	Grade 3–4	All Grades	Grade 3–4
Any AE	9 (100)	7 (77.8)	12 (100)	11 (91.7)
Treatment-related AE	9 (100)	7 (77.8)	12 (100)	8 (66.7)
Anemia	3 (33.3)	1 (11.1)	2 (16.7)	0
Neutropenia	7 (77.8)	4 (44.4)	8 (66.7)	7 (58.3)
Febrile neutropenia	0	0	2 (16.7)	2 (16.7)
Thrombocytopenia	4 (44.4)	1 (11.1)	2 (16.7)	0
Fatigue	6 (66.7)	0	8 (66.7)	0
Diarrhea	6 (66.7)	1 (11.1)	7 (58.3)	0
Nausea	5 (55.6)	0	5 (41.7)	0
Vomiting	2 (22.2)	0	3 (25.0)	0
Anorexia	4 (44.4)	0	7 (58.3)	0
Mucositis	5 (55.6)	1 (11.1)	7 (58.3)	0
Other GI toxicity	1 (11.1)	0	1 (8.3)	0
Hand-Foot syndrome	7 (77.8)	0	9 (75.0)	1 (8.3)
Rash	1 (11.1)	0	4 (33.3)	0
Pruritus	1 (11.1)	0	0	0
Other skin toxicity	2 (22.2)	0	1 (8.3)	0
Flu-like symptoms	2 (22.2)0	0	2 (16.7)	0
Fever	2 (22.2)	0	3 (25.0)	0
Infection	4 (44.4)	0	5 (41.7)	1 (8.3)
Pneumonitis	1 (11.1)	1 (11.1)	0	0
Thrombophlebitis	1 (11.1)	0	1 (8.3)	0
Cerebral infarction	0	0	1 (8.3)	0
Chest pain-cardiac	0	0	1 (8.3)	0
Pain	2 (22.2)	0	2 (16.7)	0
Edema limbs	2 (22.2)	0	2 (16.7)	0
Myalgia	1 (11.1)	0	1 (8.3)	0
Hypokalemia	1 (11.1)	1 (11.1)	1 (8.3)	1 (8.3)
Injection site reaction	0	0	2 (16.7)	0
Peripheral sensory neuropathy	1 (11.1)	0	0	0
Dysuria	0	0	1 (8.3)	0

## Data Availability

The data presented in this study are available on request from the corresponding author.

## Supplementary Materials

The following supporting information can be downloaded at: https://www.mdpi.com/article/10.3390/pharmaceutics14030509/s1, Trial protocol; Figure S1: Clustered boxplot for YKL-40 (a), IL-6 (b), CA19-9 (c) and CRP (d) through 6 months of treatment; Table S1: CONSORT 2010 checklist; Table S2: Changes in circulating biomarkers through 6 months of treatment; Table S3: EORTC QLQ-C30 Global health status and functional scales
