# Supplementary material for: A Randomized Placebo-Controlled Phase 2 Study of Gemcitabine and Capecitabine with or without T-ChOS as Adjuvant Therapy in Patients with Resected Pancreatic Cancer (CHIPAC)
Source: Pharmaceutics. 2022 Feb 25;14(3):509. doi: 10.3390/pharmaceutics14030509 (PMC8955369; doi:10.3390/pharmaceutics14030509)
Supplement: Supplementary file 1 [file pharmaceutics-14-00509-s001.zip › CHIPAC protokol v.3 20160607r.pdf]

CLINICAL STUDY PROTOCOL: CHIPAC

A Single Center, Randomized, Double-Blind, Placebo-Controlled Phase 2 Study of Gemcitabine (GEM) and Capecitabine (CAP) with or without T-ChOS™ as adjuvant therapy in patients with surgically resected pancreatic cancer

INVESTIGATIONAL PRODUCT (IP):	T-ChOS™
PROTOCOL NUMBER:	GI 1604
DATE FINAL:	June 7, 2016
EudraCT NUMBER:	2014-001163-12

SPONSOR NAME / ADDRESS:	Dorte Nielsen, Professor, MD, DMSc<br>Department of Oncology<br>Herlev & Gentofte Hospital<br>Herlev Ringvej 75<br>DK-2730 Herlev, Denmark
PRINCIPAL INVESTIGATOR NAME / ADDRESS:	Inna Chen, MD<br>Department of Oncology<br>Herlev & Gentofte Hospital<br>Herlev Ringvej 75<br>DK-2730 Herlev, Denmark
OTHER CLINICALLY RESPONSIBLE:	Benny Vittrup Jensen, MD<br>Julia S. Johansen, Professor, MD, DMSc<br>Vibeke Parner, MD<br>Department of Oncology<br>Herlev & Gentofte Hospital<br>Herlev Ringvej 75<br>DK-2730 Herlev, Denmark<br><br>Lilja Kjalarsdottir, Ph.d.<br>Johannes Gislason, Ph.d.<br>Genis<br>Arleynir 8, 112 Reykjavik, Iceland

SIGNATURE PAGE

I, the undersigned, have read and understand the protocol specified below and agree that it contains all necessary information for conducting the study. The study protocol, the Clinical Study Agreement and the additional information given will serve as a basis for co-operation in this study.

I agree to conduct the study according to protocol version 1.0 and according to the ethical principles that have their origin in the Declaration of Helsinki and that are consistent with ICH Good Clinical Practice and the applicable laws and regulations.

Signature Principal Investigator: ____

Date Signature PI

____

Name in print

Signature Sponsor: ____

Date Signature Sponsor

____

Name in print

Contact information

Sponsor: Dorte Nielsen, Professor, MD, DMSc

[redacted]

Principal Investigator: Inna Chen, MD

[redacted]

**Title**	A Single Center, Randomized, Double-Blind, Placebo-Controlled Phase 2 Study of Gemcitabine (GEM) and Capecitabine (CAP) with or without T-ChOS^{TM} as adjuvant therapy in patients with surgically resected pancreatic cancer.
**Sponsor**	Investigator initiated study. Department of Oncology Herlev & Gentofte University Hospital, Denmark.
**Rational**	The standard adjuvant treatment for patients after operation for pancreatic cancer is 6 months of GEM with oral CAP. The disease recurrence rate is high and the median disease-free survival (DFS) is about 14 months and the overall survival (OS) is 28 months. New more effective regimens in terms of inhibition of cancer relapse are needed.<br>The combination of GEM/CAP with the food supplement T-ChOS^{TM} has not been evaluated in the adjuvant treatment of patients operated for pancreatic cancer.
**Trial Design**	A 1:1 randomized, double-blind, placebo-controlled phase II design.
**Target Population**	Adjuvant treatment in patients operated for pancreatic cancer.
**Objectives**	• To compare the efficacy, safety and quality of life of GEM/CAP/T-ChOS^{TM} versus GEM/CAP/Placebo<br>• To assess whether high plasma concentrations of YKL-40 and IL-6 predict short DFS<br>• To assess whether high levels of circulating tumor *KRAS* mutations predict short DFS
**Primary end points**	Disease-free survival (DFS), defined as the time from the date of randomization to the date of disease recurrence determined by investigator assessment of objective radiographic disease assessments per Response Evaluation Criteria in Solid Tumors (RECIST) v1.1, or death, whichever is earlier.
**Secondary end points**	1) Overall survival (OS), which is defined as the time from the date of randomization to the date of death of any cause<br>2) Safety (Data on safety parameters) and tolerability of the treatment regimens assessed by a summary of adverse events and clinical laboratory assessments<br>3) Quality of life (QoL)<br><br>Exploratory<br>1) Determination of plasma YKL-40 and IL-6 and circulating tumor *KRAS* mutations in plasma
**Number of patients**	180 evaluable patients
**Number of centers**	Single center
**Selection criteria**	**Inclusion criteria**<br>1. Signed informed consent<br>2. Histologically confirmed resected ductal pancreatic adenocarcinoma with macroscopic complete resection (R0 and R1). Subjects with neuroendocrine (and mixed type) tumors are excluded<br>3. Subject should be able to start treatment no later than 12 weeks post-surgery<br>4. Male or non-pregnant, non-lactating females who are ≥18 years of age at the time of signing the informed consent form (ICF)<br>5. ECOG/WHO Performance Status (PS) 0-1<br>6. Females of child-bearing potential (defined as a sexually mature woman who (1) has not undergone hysterectomy [the surgical removal of the uterus] or bilateral oophorectomy [the surgical removal of both ovaries] or (2) has not been naturally postmenopausal for at least 24 consecutive months [i.e., has had menses at any time during the preceding 24 consecutive months]) must:

Herlev Hospital

	• Agree to the use of two physician-approved contraceptive methods (oral, injectable, or implantable hormonal contraceptive; tubal ligation; intra-uterine device; or vasectomized partner) while on study IP; and for 3 months following the last dose of IP<br>• Has negative serum pregnancy test (β -hCG) result at screening<br>7. Male subjects:<br>• Must practice true abstinence or partner (female of childbearing potential) shall agree to use an oral, injectable, or implantable hormonal contraceptive, tubal ligation or intra-uterine device during sexual contact, while patient participates in the study, during dose interruptions and for 3 months following IP discontinuation, even if he has undergone a successful vasectomy<br>8. Understand and voluntarily sign an ICF prior to any study related assessments or procedures being conducted<br>9. Be able to adhere to the study visit schedule and other protocol requirements<br>10. Acceptable hematology parameters defined as:<br>• Absolute neutrophil count (ANC) $\geq 1.5 \times 10^9$/L<br>• Platelet count $\geq 100 \times 10^9$/L<br>• Haemoglobin ≥ 5.6 mmol/L<br>11. Acceptable liver function defined as:<br>• Serum bilirubin < 1.5 x upper limit of normal (ULN)<br>• ASAT/ALAT < 2.5 x ULN<br>12. Acceptable renal function with a creatinine clearance ≥ 50 mL/min/ (e.g., using the Cockroft-Gault formula)<br><br>**Exclusion Criteria**<br>1. Prior neo-adjuvant treatment, radiation therapy, or systemic therapy for pancreatic adenocarcinoma<br>2. Presence of or history of metastatic or locally recurrent pancreatic adenocarcinoma<br>3. Other malignancies, except adequately treated basal carcinoma or squamous cell carcinoma of the skin or *in situ* cervix carcinoma or incidental prostate cancer (T1a, Gleason score ≤ 6, PSA < 0.5 ng/ml), or any other tumor with a DSF survival of ≥ 5 years<br>4. History of serious or concurrent illness or uncontrolled medical disorder; any medical condition that might be aggravated by chemotherapy treatment or which could not be controlled; including, but not restricted to:<br>• Active infection requiring antibiotics within 2 weeks before the study inclusion<br>• Concurrent congestive heart failure NYHA class III - IV<br>• Unstable angina pectoris, or myocardial infarction within 6 months and/or prior poorly controlled hypertension<br>• History of interstitial lung disease, slowly progressive dyspnea and unproductive cough, sarcoidosis, silicosis, idiopathic pulmonary fibrosis, pulmonary hypersensitivity pneumonitis or multiple allergies<br>• Concomitant use of immunosuppressive or myelosuppressive medications that would in the opinion of the investigator, increase the risk of serious neutropenic complications<br>5. Known or suspected allergy to the investigational agents or any agents given in association with this trial<br>6. Any psychological, familial, sociological, or geographical condition which does not permit protocol compliance and medical follow-up

	7. Enrollment in any other clinical protocol or investigational study with an interventional agent or assessments that may interfere with study procedures<br>8. Any condition including the presence of laboratory abnormalities, which places the subject at unacceptable risk if he/she were to participate in the study<br>9. Any condition that confounds the ability to interpret data from the study<br>10. Unwillingness or inability to comply with study procedures<br>11. Current use of anticoagulation therapy such as heparins both unfractionated and low molecular weighted
**Investigational Drugs Dose/Route/Regime**	50% of the patients will receive T-ChOS™ in combination with GEM/CAP.<br>T-ChOS: **600 mg** given p.o. (two capsules, each 300 mg) daily in the morning 30 minutes before food.<br>GEM: **1000 mg/m²** i.v. on day 1, day 8 and day 15 of every 28 day cycle for 6 cycles.<br>CAP: **1660 mg/m²/day** p.o. twice daily 21/28 day i. e. 24 weeks.
**Reference Drugs Dose/Route/Regime**	50% of the patients will receive placebo in combination with GEM/CAP.<br>Placebo: **600 mg** given p.o. (two capsules, each 300 mg) daily in the morning 30 minutes before food.<br>GEM: **1000 mg/m²** i.v. on day 1, day 8 and day 15 of every 28 day cycle for 6 cycles.<br>CAP: **1660 mg/m²/day** p.o. twice daily 21/28 day i. e. 24 weeks.
**Duration of treatment**	Subjects in both arms will receive GEM with oral CAP for 6 months, followed by continuation of the food supplement T-ChOS™/Placebo. Patients will continue treatment for a maximum of 5 years or until disease recurrence, death, unacceptable toxicity, or withdrawal of consent.
**Follow up**	Patients that are withdrawn from the study, will be followed-up at day 30 (±5 days) after last treatment. Subjects will be followed for disease recurrence and survival every third month until death.
**Sample size**	The study is designed as a randomized, double blind phase II trial, with the primary endpoint DFS. The Kaplan-Meier estimator will be used to estimate DFS and differences in DFS will be tested using the log-rank test. Treatment effect adjusted for any differences in two groups will be estimated using Cox proportional hazards regression. The median DFS after adjuvant treatment with conventional treatment is approximately 14 month. If the true hazard ratio (relative risk) of control subjects relative to the experimental subjects is 0.6 we will need to study 90 patients in the investigational arm and 90 patients in the reference arm to be able to reject the null hypothesis at a 10% significance level that the experimental and control survival curves are equal with probability (power) of 90%. With an annual accrual rate of 70 patients per year during three years and extra two years of follow-up the study is expected to generate the necessary number of events within a five year period.
**Statistical Analysis**	The primary endpoint DFS will be analyzed using survival techniques. Survival curves will be estimated using the method of Kaplan-Meier, and differences in survival times will be tested using the log-rank test. Proportional hazards regression will be used to estimate the effect of treatment on DFS. Both the unadjusted effect – a model with only treatment included – as well as the adjusted effect – a model with the stratification factors as well as treatment included - will be estimated. Results of the analyses will be presented as hazard ratios (HRs) together with confidence intervals.
**Key dates**	Anticipated start of study: June 1, 2016<br>Estimated recruitment period: June 1, 2016 – May 31, 2019<br>End of follow-up: May 31, 2021

Table of Contents

1. LIST OF ABBREVIATIONS AND DEFINITIONS OF TERMS 10
2. GENERAL INFORMATION/STUDY ADMINISTRATIVE STRUCTURE 11
2.1. PARTICIPATING CANCER CENTER 11
3. BACKGROUND 12
3.1. YKL-40 AND CANCER 12
3.2. CHITIN AND T-ChOS™ 12
3.3. BIOMARKERS IN PANCREATIC CANCER 13
3.4. RESEARCH HYPOTHESES 14
3.5. INVESTIGATIONAL PRODUCTS 14
3.5.1. T-ChOS™ (BENECTA™) 14
3.5.2. GEMCITABINE 14
3.5.3. CAPECITABINE AND GEMCITABINE 15
3.5.4. PLACEBO 15
3.6. CLINICAL STUDIES WITHINVESTIGATIONAL PRODUCTS INPANCREATIC CARCINOMA AND OTHER CANCERS 15
3.6.1. T-ChOS™ TREATMENT OF PATIENTS AND HEALTHY SUBJECTS 15
3.6.2. T-ChOS™ TREATMENT OF PATIENTS WITH CANCERS 16
4. STUDY RATIONAL 16
4.1. RATIONALE FOR CLINICAL DOSE 16
5. STUDY OBJECTIVES 17
5.1. PRIMARY OBJECTIVE 17
5.2. SECONDARY OBJECTIVES 17
5.3. EXPLORATORY OBJECTIVES 18
6. STUDY ENDPOINTS 18
6.1. PRIMARY ENDPOINT 18
6.2. SECONDARY ENDPOINTS 18
6.3. EXPLORATORY ENDPOINTS 18
7. STUDY DESIGN 18
7.1. STUDY OUTLINE 18
8. STUDY ASSESSMENTS AND PROCEDURES 19
8.1. ASSESSMENTS PER VISIT 19

8.2. SCHEDULE OF EVENTS 22
8.3. FOLLOW-UP PERIOD ASSESSMENT 23
8.4. BIOMARKER TESTING AND BIOBANK 23
9. SELECTION AND WITHDRAWAL OF SUBJECTS 23
9.1. GENERAL ELIGIBILITY CRITERIA 23
9.2. SCREENING AND ENROLMENT OF SUBJECTS 24
9.3. SUBJECT INCLUSION CRITERIA 24
9.4. SUBJECT EXCLUSION CRITERIA 24
9.5. CONTINUATION CRITERIA 25
9.6. WITHDRAWAL OF SUBJECTS 25
9.7. CONSIDERATIONS AFTER WITHDRAWAL 26
10. TREATMENT OF SUBJECTS 26
10.1. TREATMENT ADMINISTRATION AND TREATMENT MODIFICATIONS 26
10.1.1. PRE-DEFINED DOSAGE SCHEDULE 26
10.1.2. INDIVIDUAL DOSE MODIFICATIONS 26
10.1.3. DOSE DELAY 29
10.2. EXPECTED, MANAGEABLE TREATMENT RELATED TOXICITY 29
10.2.1. LIST OF COMMON AND EXPECTED TREATMENT RELATED TOXICITY 30
10.3. CONCOMITANT THERAPY 30
10.3.1. RECOMMENDED CONCOMITANT TREATMENTS 30
10.3.2. PROHIBITED AND RESTRICTED THERAPIES DURING THE STUDY 31
10.3.3. SUPPORTIVE AUTHORIZED TREATMENTS 31
10.4. COMPLIANCE WITH THE TREATMENT 31
10.5. PACKAGING AND LABELLING OF INVESTIGATIONAL PRODUCTS 31
10.6. BLINDING 31
11. ASSESMENT OF EFFICACY 32
11.1. TUMOUR ASSESMENT 32
12. ASSESSMENT OF SAFETY 32
12.2. BLOOD PRESSURE AND HEART RATE 32
12.3. ELECTROCARDIOGRAM (ECG) 33
12.4. ADVERSE EVENTS (AE) 33
12.4.1. METHODS FOR ELICITING, RECORDING AND FOLLOW-UP OF ADVERSE EVENTS 33
12.4.2. SEVERITY OF ADVERSE EVENTS 33

12.4.3. ATTRIBUTION DEFINITIONS ... 34
12.4.4. FINAL OUTCOME OF EVENT ... 34
12.4.5. DEFINITION OF AN UNEXPECTED ADVERSE EVENT ... 34
12.4.6. DEFINITION OF SERIOUS ADVERSE EVENTS (SAE) ... 34
12.5. ADVERSE REACTIONS (AR) ... 35
12.5.1. DEFINITION OF ADVERSE REACTION (AR) ... 35
12.5.2. UNEXPECTED ARVERSE REACTION (UAR) ... 35
12.5.3. SERIOUS ADVERSE REACTION (SAR) ... 35
12.5.4. DEFINITION OF SUSPECTED UNEXPECTED SERIOUS ADVERSE REACTION (SUSAR) ... 36
12.6. REPORTING OF SERIOUS ADVERSE EVENT (SAE) AND SUSPECTED UNEXPECTED SERIOUS ADVERSE REACTION (SUSAR) ... 36
12.7. FOLLOW-UP OF SERIOUS ADVERSE EVENTS ... 36
12.8. ANNUAL SAFETY REPORT ... 36
12.9. TRIAL SAFETY COMMITTEE ... 36
13. ASSESSMENT OF QUALITY OF LIFE ... 36
14. STATISTICS AND DATA MANAGEMENT ... 36
14.1. DATA MANAGEMENT ... 36
14.1.1. DATA ENTRY AND DATA VALIDATION ... 36
14.1.2. CODING ... 37
14.2. STATISTICAL ANALYSIS ... 37
14.2.1. OBJECTIVES AND ENDPOINTS ... 37
14.2.2. ANALYSES ... 37
14.3. RANDOMIZATION AND STRATIFICATION OF SUBJECTS ... 38
15. TRANSLATIONAL ANALYSES ... 38
16. DIRECT ACCESS TO SOURCE DATA/DOCUMENTS ... 38
17. QUALITY CONTROL AND QUALITY ASSURANCE ... 39
17.1. SOURCE DATA ... 39
17.2. STUDY MONITORING ... 39
18. ETHICS ... 39
18.1. INDEPENDENT ETHICS COMMITTEE ... 39
18.2. ETHICAL CONDUCT OF THE STUDY ... 40
18.3. PATIENT INFORMATION AND INFORMED CONSENT ... 41
19. DATA HANDLING AND RECORD KEEPING ... 42

19.1. CASE REPORT FORMS 42
19.2. RECORD KEEPING 42
20. INSURANCE 42
21. PUBLICATION POLICY 42
22. ECONOMY 43
23. SUPPLEMENTS 43
23.1. CHANGES OF THE STUDY PROTOCOL 43
23.2. APPLICATION TO REGULATORY AUTHORITIES AND ETHICS COMMITTEE ... 43
23.3. STAFF INFORMATION 43
23.4. CRITERIA FOR TERMINATION OF THE STUDY 44
23.5. STUDY TIME SCHEDULE 44
24. REFERENCES 45
25. COORDINATING PRINCIPAL INVESTIGATOR'S AGREEMENT 49
26. PRINCIPAL INVESTIGATOR'S AGREEMENT 49
27. APPENDICES 50
27.1. APPENDIX: PROTOCOL SUMMARY (IN DANISH), VERSION 1.0, 2015-10-21 51
27.2. APPENDIX: PATIENT INFORMATION SHEET (IN DANISH), VERSION 1.0, 2015-10-21 56
27.3. APPENDIX: INFORMED CONSENT (IN DANISH), VERSION 1.0, 2015-10-21 65
27.4. APPENDIX: FIRST CONTACT AND INFORMED CONSENT GUIDANCE (IN DANISH), VERSION 1.0, 2015-08-20 67
27.5. NOTIFICATION OF SERIOUS ADVERSE EVENT (SAE) 68
27.6. APPENDIX: EORTC QUALITY OF LIFE QUESTIONNAIRE C30 (QLQ-C30) VERSION 3.0 68

1. LIST OF ABBREVIATIONS AND DEFINITIONS OF TERMS

AE	Adverse Event
CAP	Capecitabine
CRF	Case Report Form
CT	Computerized Tomography
ECG	Electrocardiogram
FU	Follow-Up
GCP	Good Clinical Practice
GEM	Gemacitabine
GFR	Glomerular Filtration Rate
IEC	Independent Ethics Committee
OS	Overall Survival
PFS	Progression Free Survival
PD	Progressive Disease
PS	Performance Status
DFS	Disease Free Survival
SAE	Severe Adverse Event
SD	Stable Disease
SUSAR	Suspected Unexpected Serious Adverse Reactions
QoL	Quality of Life

2. GENERAL INFORMATION/STUDY ADMINISTRATIVE STRUCTURE

SPONSOR:	Dorte Nielsen, Professor, MD DMSc<br>Department of Oncology<br>Herlev & Gentofte Hospital<br>Herlev Ringvej 75, 2730 Herlev, Denmark<br>[redacted]
PRINCIPAL INVESTIGATOR:	Inna Chen, MD<br>Department of Oncology<br>Herlev & Gentofte Hospital<br>Herlev Ringvej 75, 2730 Herlev, Denmark<br>[redacted]
CLINICAL STUDY MONITOR	Clinical Trial Unit/Klinisk Forskningsenhed (KFE)<br>Department of Oncology<br>Herlev & Gentofte Hospital<br>Herlev Ringvej 75, 2730 Herlev, Denmark
DATA MANAGER	Clinical Trial Unit/Klinisk<br>Forskningsenhed (KFE)<br>Department of Oncology<br>Herlev & Gentofte Hospital<br>Herlev Ringvej 75 2730 Herlev, Denmark
STATISTICIAN	Christian Dehlendorff, Ph.d.<br>Senior statistician at Danish Cancer Society<br>Research Center<br>Strandboulevarden 49<br>2100 København Ø, Denmark<br>[redacted]
MONITORING	GCP unit at Copenhagen University Hospital<br>Bispebjerg Hospital, Building 51, 3rd floor<br>Bispebjerg Bakke 23<br>2400 København NV Denmark<br>[redacted]

2.1. PARTICIPATING CANCER CENTER

Department of Oncology
Herlev& Gentofte University Hospital
Herlev Ringvej 75,DK-2730 Herlev, Denmark

3. BACKGROUND

Worldwide 337,872 people were registered in 2012 with pancreatic cancer (PC) (1). The incidence is increasing and patients with PC have the highest mortality rate. PC was the 7th most common cause of death from cancer with 330,372 deaths registered in 2012; it is the 4th-leading cause of cancer related death in the United States (~41.780) and Europe (~75.000) (1-3). The number of new cases per year in Denmark (incidence 2008-2012) is ~950, with prevalence increasing with increasing age (NORDCAN 2014), and equally distributed between the two sexes. The prognosis has only improved slightly the last decade and despite the enthusiastic efforts of the last years, the 5-year survival rate remains at only ~5%.

Only ~20% of patients with PC can be operated with curative intent, since most patients have locally advanced or metastatic PC at the time of diagnosis (4, 5). However, even after radical resection, the recurrence rate is very high, and the majority of the patients relapse within two years. Based on CONCO 001, ESPAC-1, ESPAC-3 and ESPAC-4 trials, all patients with resected PC are recommended adjuvant treatment; combination of gemcitabine and capecitabine is preferred due to its efficacy and acceptable toxicity profile (6-9). Unfortunately, the prognosis for patients with PC after resection and adjuvant gemcitabine chemotherapy is very disappointing; with median disease-free survival (DFS) of only 14 months (6). The median overall survival (OS) of only 28 months and the 5-year OS of approximately 29% can be achieved after adjuvant combination of gemcitabine with capecitabine (9). Thus, there is a significant, unmet medical need for new treatments of patients with PC.

3.1. YKL-40 AND CANCER

YKL-40 (also named chitinase 3 like-1 protein, CHI3L1), is a member of the mammalian chitinase like proteins and is a highly conserved glycoprotein. YKL-40 is mainly produced by cancer cells (including PC), macrophages and neutrophils (10). YKL-40 binds chitin, heparin and collagen, but has no chitinase activity. YKL-40 regulates vascular endothelial growth factor (VEGF) and has a role in inflammation and angiogenesis (10-14), cell proliferation and differentiation (15), remodeling of the extracellular matrix(10), activates Akt signaling in colonic epithelial cells (16), protects against apoptosis (17) and promote tumor progression (18). YKL-40 regulates cellular and tissue responses via IL-13 receptor α2 (19).

It has been suggested that YKL-40 is a new therapeutic target for patients with cancer, since antibodies against YKL-40 could inhibit tumor angiogenesis and cancer progression (13, 14).

3.2. CHITIN AND T-ChOS™

T-ChOS™ is a technical name for a food supplement product that is marketed in Iceland under the name Benecta™. T-ChOS™ is manufactured using shellfish derived chitin as a raw material and is produced by Genis in Iceland. T-ChOS™ a blend of chitooligosaccharides that are hetero-complexes of N-acetyl-glucosamine and D-glucosamine. This is an optimized blend where chitooligosaccharides with low bioactivity have been sorted out. T-ChOS™ chitooligasaccharides have been specially selected to have high bioactivity in inflammatory models and strong binding affinity to YKL-40. Data from Genis show that T-ChOS™ has anti-inflammatory and tissue protective effect on cartilage and bone tissue in animal models of osteoarthritis, osteoporosis and rheumatoid arthritis. T-ChOS™ also increases the chemo-sensitivity of cancer cells *in vitro*. Chitin derivatives have been found to have exceptionally low toxicity *in vivo* and *in vitro* and are therefore considered very safe for consumption. The bioactivity of chitooligosaccharides has been the topic of several preclinical studies in the cancer field because of their

ability to reduce chronic inflammation and stimulate immune system function. To summarize these findings, *in vivo* treatment of mammary tumor-bearing mice with chitin promoted immune effector functions, inhibited tumor growth and development of lung metastasis (20); chitohexaose and N-acetyl-chitohexaose were shown to promote regression of tumor growth in a sarcoma 180 mouse model (21) and in a Meth-A solid tumor model (22); in a Lewis lung carcinoma N-acetyl-chitohexamer showed anti-metastatic and tumor growth-inhibitory activity (23) and another publication using a chitooligomer blend reproduced these results (24); various low molecular weight chitosan blends have shown anti-tumor effects and this activity has been ascribed to increased natural killer cell activity in the tumor environment(25). Furthermore, a study using an Ehrlich ascites tumor model showed potent anti-angiogenic activity of chitosan oligomers(26).

3.3. BIOMARKERS IN PANCREATIC CANCER

Substantial gap exists in our knowledge of this serious disease, mainly because PC is a complex heterogeneous disease distinguished by a landscape of gene mutations (27, 28); this can be one of the reasons to lack of prognostic or predictive biomarkers identified in patients with PC at present. The concentration in plasma of the protein CA 19-9 is the most widely used biomarker for assessing the disease burden, monitoring of disease recurrence and prognosis (29). However, it is not possible to use plasma CA 19-9 in a number of patients who do not express Lewis antigen (30), and plasma CA 19-9 can also increase due to benign disorders of the pancreas, in particular chronic inflammation, cholestasis, and jaundice (31, 32). Thus, there is a great need for new reliable biomarkers for use in the daily practice in patients with PC.

Approximately 70% of patients with metastatic PC have elevated plasma YKL-40 compared to age-matched healthy subjects and high plasma YKL-40 in patients with cancer, including patients with PC, are associated with short OS (10, 33). Preoperative plasma YKL-40 is elevated in ~30% of patients with stage I and II PC (33). Studies have shown that plasma YKL-40 is a new prognostic biomarker and independent of serum CA 19-9 (10, 33).

There is a growing interest in liquid biopsy, based on a simple blood or urine draw. Dying cancer cells shed DNA, circulating tumor DNA (ctDNA), which makes possible to detect mutations when surgical biopsy is not available/evaluable. Detecting of ctDNA in blood and urine in patients with malignancies is actively under investigation and deemed to be a promising innovative approach for early detection, treatment monitoring, tracking emergent mutations while patients undergo therapy and identifying an acquired resistance to treatment, as well as sampling heterogeneous tumors (34, 35). The majority of PC has *KRAS* mutations (36, 37). CtDNA *KRAS* mutations have been shown to be measurable in plasma and have been proven useful as diagnostic and prognostic biomarkers in patients with cancer. We have recently demonstrated that pretreatment plasma CA 19-9 in combination with ctDNA *KRAS* mutation load in 182 unresectable patients with PC (Johansen, J. et al: ASCO 2015, Abstract 288) was found as a strong prognostic factor.

Several reports have highlighted the integral role of interleukin-6 (IL-6) in facilitating inflammation cascade, key pathways and processes within the respective tumor microenvironment (38-42). Inflammation plays an important role in cancer progression (43-49). IL-6, an immune-modulating cytokine, is produced by the PC cells, macrophages, lymphocytes, and endothelial cells and regulates inflammation, development of cachexia and the stromal desmoplasia (50). IL-6mediated inflammation may contribute to cluster debilitating symptoms like anorexia, weight loss, fatigue, anaemia, fever, pain and depression, and associated with poorer outcome (51-53). Greater activation of the systemic inflammatory response and high plasma IL-6 levels in more than 60% of patients with advanced PC have been reported; and higher IL-6 levels correlate with short OS(33, 50, 54-57). Rapid weight loss and

cachexia is a major problem for many patients with PC and is associated with reduced survival and quality of life (58). IL-6 stimulates YKL-40 production and there is a strong correlation between plasma levels of IL-6 and YKL-40.

3.4. RESEARCH HYPOTHESES

I. Time to disease relapse and death can be extended and the quality of life can be improved in patients with PC with addition of T-ChOS™ to a combination with standard adjuvant chemotherapy with gemcitabine and capecitabine.

II. Analysis of ctDNA *KRAS* mutation load in the blood and plasma YKL-40 and IL-6 from patients with PC can be used for better assessment of the patients, the treatment efficacy, disease relapse and prognosis.

3.5. INVESTIGATIONAL PRODUCTST-ChOS™ (BENECTA™)

T-ChOS™ (marketed food supplement product in Iceland under the name Benecta™) is composed of two mono sugar moieties; N-acetyl glucosamine and glucosamine. The T-ChOS™ product consists of a mixture of oligosaccharides with degrees of polymerization (DP) in the range of DP3-25, whereof the most abundant compositions are in the range of DP5-15. These oligomers have been specially selected because of their high affinity to YKL-40. The internal part of the molecules is comprised mostly of sequences where two N-acetyl glucosamine moieties are separated by a glucosamine moiety. This is an important feature increasing the resistance to hydrolysis specifically catalyzed by human family 18 chitinases.

Genis has performed several preclinical studies in order to assess efficacy of T-ChOS™ in severe degenerative and inflammatory diseases, both *in vitro* and *in vivo*. This includes studies performed in cell models involving various types of human cells, bovine and human cartilage cultures and aggressive disease models in rats. The overall results from these studies indicate that T-ChOS™ alleviates inflammation and contributes to protecting tissues in inflammatory joint diseases. Additionally, no adverse reactions have been observed in preclinical studies using T-ChOS™. This supports the safety profile seen with chitin and chitosan related products in the literature (59). Moreover, studies on carcinoma cell lines indicate increased chemo-sensitivity with T-ChOS™.

There are no published studies of patients with PC treated with T-ChOS™. However, the studies from the literature suggest that chitooligosaccharide induced blockade of YKL-40 signaling pathways may represents a promising approach in combination with chemotherapy in patients operated for PC as a first line.

3.5.2.GEMCITABINE

Chemically gemcitabine is a nucleoside analog in which the hydrogen atoms on the 2' carbon of deoxycytidine are replaced by fluorine atoms. As with fluorouracil and other analogues of pyrimidines, the triphosphate analogue of gemcitabine replaces one of the building blocks of nucleic acids, in this case cytidine, during DNA replication (60).The process arrests tumor growth, as only one additional nucleoside can be attached to the "faulty" nucleoside, resulting in apoptosis. Another target of gemcitabine is the enzyme ribonucleotide reductase (RNR). The diphosphate analogue binds to RNR active site and inactivates the enzyme irreversibly. Once RNR is inhibited, the cell cannot produce the deoxyribonucleotides required for DNA replication and repair, and cell apoptosis is induced (61).

Gemcitabine is widely used in many different types of cancer, e.g. non-small cell lung cancer, transitional carcinoma of the bladder, ovarian cancer, breast cancer, and esophageal cancer. Gemcitabine alone or in combination with capecitabine is indicated in patients with resected PC as an adjuvant treatment (6, 8, 9).

3.5.3.CAPECITABINE AND GEMCITABINE

Capecitabine is administered as a non-cytotoxic, systemic prodrug of 5'-deoxy-5-fluorouridine. After administration it is mainly absorbed unchanged from the gastrointestinal tract and sequentially converted to the cytotoxic 5-fluorouracil (5-FU) via a number of metabolic steps. The final reaction of this signaling pathway is catalyzed by thymidine phosphorylase (TP). 5-FU is preferably generated at the tumor site through the use of higher concentrations of TP present in the tumor tissue compared to normal tissue.

Capecitabine in combination with gemcitabine has been evaluated in the ESPAC-4 trial for resected pancreatic cancer (9). ESPAC-4 aimed to determine whether combination chemotherapy improved survival compared to monotherapy with gemcitabine. Patients with pancreatic ductal adenocarcinoma were randomized to have either six 4 week cycles of IV gemcitabine alone or gemcitabine with oral capecitabine. Median survival (months) for patients treated with combination was 28.0 (95% CI, 23.5 – 31.5) and 25.5 (22.7 – 27.9) for monotherapy. Stratified log-rank analysis revealed an HR=0.82 [95% CI, 0.68 – 0.98]; $\chi2$ (1) = 4.61, P=0.032. 196 out of 366 gemcitabine patients in the safety set reported 481 grade 3/4 adverse events, while 226 out of 359 combination patients reported 608 grade 3/4 adverse events (P=0.242).

Based on these results, all patients with resected PC are recommended adjuvant treatment with combination of gemcitabine and capecitabine due to its efficacy and acceptable toxicity profile (9).

3.5.4.PLACEBO

Matching placebo capsules for T-ChOS™ (300 mg) will be provided by Genis. The placebo capsules will contain maltodextrin oligosaccharides and will be packed in the same capsules as T-ChOS™. There are no inactive ingredients or excipients in T-ChOS™ and therefore the same will apply to maltodextrin. The contents of the labels will be in accordance with all applicable regulatory requirements.

3.6. CLINICAL STUDIES WITHINVESTIGATIONAL PRODUCTS INPANCREATIC CARCINOMA AND OTHER CANCERS

3.6.1.T-ChOS™ TREATMENT OF PATIENTS AND HEALTHY SUBJECTS

There are no published studies of patients with cancer treated with T-ChOS™.
A randomized, double blind, placebo controlled trial of the effects of T-ChOS™ on patients with mild to moderate osteoarthritis (OA) of the knee was performed in 2012 by Instituto de Investicacion Clinica de Occidente (IICO) in Zapopan, Jalesco, Mexico. Subjects were randomly recruited after a diagnosis of mild or moderate OA. For outcome evaluation patient data collection form was used (Western Ontario McMaster Universities Arthritis index (WOMAC) of osteoarthritis questionnaires and Visual Analog Scale (VAS)). Patients received two 350 mg capsules (700 mg) orally once daily. Fifty four patients were enrolled and out of those, 33 were screened. Twenty six patients completed the study; 14 from the

T-ChOS™ group and 12 from the placebo group. Twenty four subjects reported adverse events; 11 from the T-ChOS™ group and 13 from the placebo group. Six subjects discontinued the study due to adverse events; 3 from the T-ChOS™ group and 3 from the placebo group. None of these adverse events were graded as serious and none of them were found to be directly related to T-ChOS™.

Table 1: *Summary of safety profile of 700 mg daily dose of* T-ChOS™ *in patients as a single agent; most relevant grade adverse events (AE) (below):*

**Group**	**No of participants**	**No of subjects reporting AE**	**% of subjects reporting AE**	**No of subjects reporting no AE**	**Subjects that discontinued due to AE**
**T-ChOS™**	16	11	69%	5	3
**Placebo**	17	13	76%	4	3
**Total**	**33**	**24**	**73%**	**9**	**6**

3.6.2. T-ChOS™ TREATMENT OF PATIENTS WITH CANCERS

Combination of T-ChOS™ in combination with chemotherapy has only been tested in few patients with cancer.

The general experience of individuals consuming T-ChOS™ has been positive. No adverse reactions have been reported to Genis and majority of individuals receiving the product have reported positive subjective experience. In 3 patients diagnosed with prostate cancer, serum PSA values have significantly gone down after few weeks of consuming 600 – 750 mg of T-ChOS ™ per day and all of them are stable and have remained progression free even though one of them was also diagnosed with bone metastasis. One patient with PC started to consume 700 mg/day T-ChOS™ after being diagnosed with pancreatitis. Shortly thereafter, the patient was diagnosed with early stage PC. After consuming T-ChOS™ together with chemo and radiation therapy, the disease became stable with no detectable tumor. Other two patients with PC had more severe disease when they started to take T-ChOS™. These two patients survived for two years and had unusually good quality of life compared to other end stage PC patients. A few other patients with multiple myeloma, glioblastoma and colon cancer have reported increased quality of life after starting consumption of T-ChOS.

4. STUDY RATIONAL

For patients operated for PC gemcitabine in combination with capecitabine is a standard adjuvant treatment (9). The development of new effective treatment strategies remains a major challenge.
High levels of YKL-40 in patients with cancer, including PC, are associated with short OS (10, 33). Thus, we believe that blockade of YKL-40-regulated signaling pathways represents a promising approach in combination with gemcitabine. A regimen of the food supplement T-ChOS™ and gemcitabine with capecitabine is thus a new option for adjuvant treatment of patients after operation for PC.

4.1. RATIONALE FOR CLINICAL DOSE

Genis has conducted various preclinical experiments in disease models that have high expression of YKL-40; these include osteoarthritis, rheumatoid arthritis, osteoporosis and cancer models. All these models have an underlying inflammation that is reflected by elevation in circulating YKL-40. Results from arthritis animal studies suggest that T-ChOS™ provides beneficial effects, mainly by reducing inflammatory symptoms, thereby sustaining tissue integrity by minimizing tissue destruction, promoting tissue regeneration and preventing scar tissue generation. Results from osteoporosis model show that T-ChOS can reduce bone destruction. Finally, results from an Ehrlich Ascites cancer model in mice suggest that oral T-ChOS™ treatment results in enhanced integrity of epithelial phenotype of the cancer cells, compared to untreated control.

Careful dose range studies have been conducted in these models, ranging from 3 mg/kg to 28 mg/kg. Remarkably a 7-8mg/kg (490-560 mg/70 kg) dose was the most effective for all diseases studied despite their variety. By extrapolating the dose curve it can be estimated that doses up to 10 mg/kg would also able to alleviate symptoms. These preclinical models have shown dose response curves that reach optimum at 7-10 mg/kg (490-700 mg/70 kg). If the dose is substantially increased from the observed optimum in these models, efficacy will gradually decrease.

Similar results are seen in cell culture studies. In cartilage tissue cultures, integrity of cartilage tissue is prolonged in cultures treated with T-ChOS™. This is associated with onset of collagen II synthesis in the tissue cultures. In cell cultures T-ChOS™ shows increased cell growth in chondrocyte cultures and increased chemo-sensitivity in various cancer cell lines. These preclinical models have shown dose response curves that reach optimum at 50-100µg/ml in all cell models. Similar to animal data, if the dose is substantially increased from the observed optimum in these models, efficacy will gradually decrease.
Anecdotal data from extensive human use of T-ChOS™, by individuals with various diagnoses such as osteoarthritis, rheumatoid arthritis, prostatitis, enlarged prostate, prostate cancer, pancreatic cancer, lung cancer and multiple sclerosis we have seen alleviation of symptoms at dosage levels between 300 and 750 mg/day. However, overwhelming majority uses 600 mg/day and Genis has stopped manufacturing the 750mg capsules. The subjects ingesting 600mg/day with good results have a bodyweight of 50-130kg. Only in mild cases is 300mg/day enough to alleviate symptoms. Similar to preclinical results ingesting more than the recommended daily dose, which is now 600mg, symptoms start reappearing.
Taking all these observations into consideration and since pancreatic cancer is generally associated with extremely high expression levels of YKL-40, we have concluded to select the upper limit of the dose range anecdotally suggested to be effective in humans; oral dose of 600 mg/day.

5. STUDY OBJECTIVES

5.1. PRIMARY OBJECTIVE

- To compare the disease free survival (DFS) (independently assessed) per RECIST 1.1 of T-ChOS™ + GEM/CAP versus placebo + GEM/CAP in patients operated for PC.

5.2. SECONDARY OBJECTIVES

- To evaluate the overall survival (OS)
- To assess the safety and toxicity of T-ChOS™ + GEM/CAP versus placebo + GEM/CAP
- To investigate and compare quality of life during treatment with T-ChOS™ + GEM/CAP versus placebo + GEM/CAP, respectively

5.3. EXPLORATORY OBJECTIVES

- To investigate whether circulating tumor *KRAS* mutations in plasma are associated with disease outcome
- To assess whether YKL-40 and IL-6 in plasma is correlated with clinical outcome

6. STUDY ENDPOINTS

6.1. PRIMARY ENDPOINT

- Diseasefree survival (DFS) according to RECIST 1.1

6.2. SECONDARY ENDPOINTS

- Overall survival (OS)
- Data on safety (Serious Adverse Events, Adverse Events leading to premature withdrawal)
- Data on Quality of Life (Quality of Life Questionnaire C30 (QLQ-C30) Version 3.0)

6.3. EXPLORATORY ENDPOINTS

- Identification of circulating tumor*KRAS* mutations in plasma
- Assessing of plasmaYKL-40 and IL-6

7. STUDY DESIGN

7.1. STUDY OUTLINE

This is a single center, randomized, double-blind, placebo-controlled phase II trial. Blinded design is reasonable due to the same backbone chemotherapy-regimen and not significant toxicity profile due to T-ChOS™. The same dose modification schedules for chemotherapy drug (see Chapter 10.1) and with the same recommended and prohibited concomitant treatments (see Chapter 10.3).

A screening visit will be performed within 4 weeks before the first study drug administration.

A patient is entering the study when all of the inclusion criteria and none of the exclusion criteria are fulfilled.

Adverse events occurring during the screening period will be recorded as an updated baseline status.
The patients are randomized into two treatment arms.

Investigational Arm: 50% of the patients will receive T-ChOS™ in combination with GEM/CAP.
T-ChOS™: **600 mg** daily p.o.
GEM: 1000 mg/m² i.v. on day 1, day 8 and day 15 of every 28 day cycle for 6 cycles.
CAP: 1660 mg/m²/day p.o. twice daily 21/28 day i. e. 24 weeks.

Reference Arm: 50% of the patients will receive placebo in combination with GEM/CAP.
Placebo: **600 mg** daily p.o.
GEM: 1000 mg/m² i.v. on day 1, day 8 and day 15 of every 28 day cycle for 6 cycles.
CAP: 1660 mg/m²/day p.o. twice daily 21/28 day i. e. 24 weeks.

Subjects in both arms will receive GEM/CAP for 6 months, followed by continuation of T-ChOS/Placebo. Patients will continue treatment for a maximum of 5 years or until disease recurrence, death, unacceptable toxicity, or withdrawal of consent.

Patients that are withdrawn from the study, will be followed-up at day 30 (±5 days) after last treatment. Subjects will be followed for disease recurrence and survival every 3 months the first year, every 4 months the second and third year, and every 6 months up to five years.

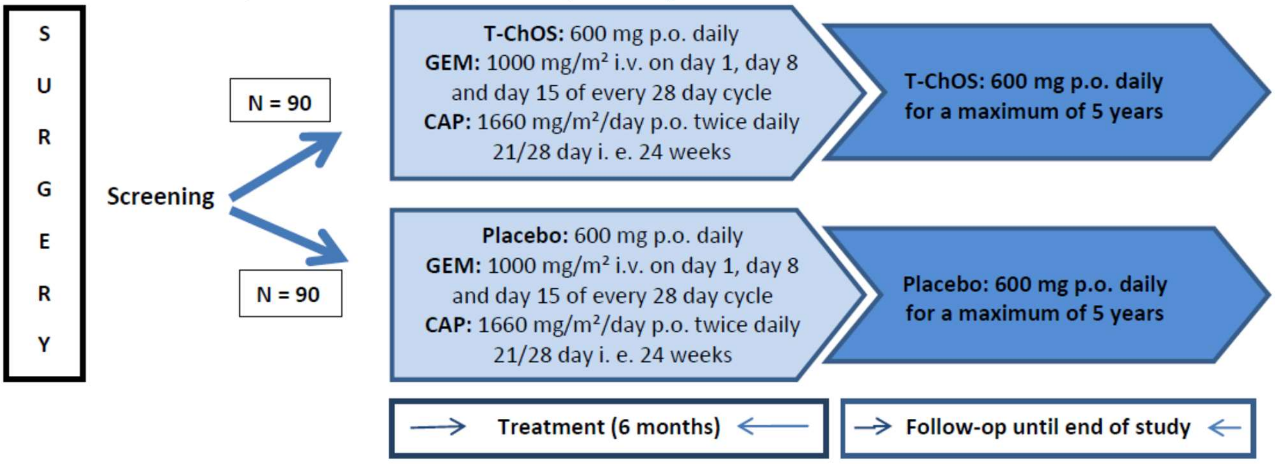

If any symptoms need to be followed up the patient will be scheduled for an additional appointment as soon as possible.

Tumor recurrence according to RECIST 1.1 criteria will be assessed every 3rd treatment cycle.

8. STUDY ASSESSMENTS AND PROCEDURES

8.1. ASSESSMENTS PER VISIT

Screening visit	Collection of written Informed consent
	Written Informed Consent to BIOPAC
(Within 28 days before first	Medical/surgical history
Study drug administration)	Physical examination
	Inclusion and exclusion criteria
	Height
	Weight
	Blood pressure, heart rate
	Electrocardiogram (ECG)
	ECOG/WHO

	<u>Blood/plasma analysis:</u>
	Haematology[1)]
	Electrolytes (including creatinine) [2)]
	Hepatic function[3)]
	CRP, Albumin, CA 19-9
	Concomitant medication
	Pregnancy test, if applicable
	Tumor assessment–radiology (baseline CT thorax/abdomen)
	- Within 28 days before first study drug administration
Day -1<br>(≤72 h before first cycle)	Medical/surgical history (i.e. update baseline status)
	Concomitant medication (i.e. update baseline status)
	Physical examination
	Weight
	Blood pressure, heart rate
	ECOG/WHO
	Common toxicity criteria (CTC) registration / Adverse event
	<u>Blood/plasma analysis:</u>
	Haematology[1)]
	Electrolytes (including creatinine)[2)]
	Hepatic function[3)]
	CRP, Albumin, CA 19-9
	Blood samples for translational analyses, YKL-40, IL-6, ct*KRAS*[4)]
	QLQ-C30 v 3.0
	Administration of study drugs
Day 8 (= day 28)<br>(Day of drug admin)	<u>Blood/plasma analysis:</u>
	Haematology[1)]
	Adverse event
	Administration of study drugs
Day 15 (= day 28)<br>(Day of drug admin)	<u>Blood/plasma analysis:</u>
	Haematology[1)]
	Adverse event
	Administration of study drugs
Day 28<br>(≤72 h before drug admin)	Weight
	Blood pressure, heart rate
	ECOG/WHO
	<u>Blood/plasma analysis:</u>

Haematology[1)]

Electrolytes (including creatinine)[2)]

Hepatic function[3)]

CRP, Albumin, CA 19-9

Blood samples for translational analyses, YKL-40, IL-6, ct*KRAS*[4)]

Concomitant medication

Adverse event

Continuation criteria

Tumor assessment – radiology (CT thorax/abdomen) (every 12weeks/3nd cycle)

QLQ-C30 v3.0 (every 12weeks/3nd cycle)

Administration of study drugs

Extra visit	Only relevant assessments should be performed:<br>Physical examination<br>Weight<br>Blood pressure, heart rate<br>ECOG/WHO<br><u>Blood/plasma analysis:</u><br>Haematology[1)]<br>Electrolytes (including creatinine)[2)]<br>Hepatic function[3)]<br>CRP, Albumin, CA 19-9<br>Blood samples for translational analysis, YKL-40, IL-6, ct*KRAS*[4)]<br>Concomitant medication<br>Adverse event	
Follow-up visit<br>(Day 30 ± 2 after last treatment administration)	Physical exam<br>Weight<br>Blood pressure/heart rate<br>ECOG/WHO PS<br>Concomitant medication<br><u>Blood/plasma analysis:</u><br>Haematology[1)]<br>Electrolytes (including creatinine)[2)]<br>Hepatic function[3)]<br>CRP, Albumin, CA 19-9<br>Blood samples for translational analysis, YKL-40, IL-6, ct*KRAS*[4)]<br>Concomitant medication<br>Adverse event<br>Tumor assessment – radiology (CT thorax/abdomen)	every 3 months the first year, every 4 months the second and third year, and every 6

months up to five years.

QLQ-C30 v3.0

8.2. SCHEDULE OF EVENTS

The schedule of events will be repeated each treatment cycle until any discontinuation criteria is met. A summary of study events to be performed at each treatment cycle is presented in the Table 2 below:

**Study parameter**	**Screening** Day ≤ -28	**Day -1** ≤72 h prior 1st dose	**Day 8** Drug admin.	**Day 15** Drug admin.	**Day 28** ≤72 h prior to admin.	**Extra visit**[8)]	**Follow-Up visit**[9)]
Informed consent	X						
Informed Consent to BIOPAC	X						
Medical/surgical history	X	X[5)]					
Inclusion/exclusion criteria	X						
Physical exam	X	X				X	X
Height	X						
Weight	X	X			X	X	X
Blood pressure/heart rate	X	X			X	X	X
Electrocardiogram (ECG)	X						
ECOG/WHO PS	X	X			X	X	X
Concomitant medication	X	X[5)]			X	X	X
Pregnancy test (if applicable)	X						
Tumor assessment – CT thorax/abdomen	X				X[6)]		X[11)]
Haematology[1)]	X	X	X	X	X	X	X
Electrolytes (including creatinine[2)])	X	X			X	X	X
Hepatic function[3)]	X	X			X	X	X
CRP, Albumin	X	X			X	X	X
CA 19-9		X			X	X	X
Blood samples (YKL-40, IL-6, ct*KRAS*[4)])		X			X	X	X
Adverse event		X	X	X	X	X	X
QLQ-C30 v3.0		X			X[7)]		X
Continuation criteria[10)]					X		

1) Haematology: Hb, Platelets, WBC including neutrophils
2) Electrolytes: P-Na, P-K, P-Ca, P-creatinine
3) Hepatic function: P-ASAT, P-ALAT, P-bilirubin, P-LD, P-ALP, coag.factor II, VII and X, INR
4) Blood samples for translational analyses, YKL-40,IL-6 and ct*KRAS* according.to BIOPAC.
5) Prior to cycle 1: Update baseline status
6) Tumor assessment – radiology (CT/MR): every 12 weeks/3nd cycle
7) Assessment of QoL – QLQ-C30 v3.0 every 12 weeks/3nd cycle
8) Assessments according to the relevance on a case to case basis
9) Follow-up visit: day 30±5 days after last treatment administration
10)Continuation criteria: Se chapter 9.5
11) Tumor assessment – radiology (CT/MR): every 3 months the first year, every 4 months the second and third year, and every 6 months up to five years.

8.3. FOLLOW-UP PERIOD ASSESSMENT

The follow-up period is the time from 30 days after the last study treatment administration until death or decision for study closure. For patients who discontinued the study due to recurrence of PC, survival information will be collected approximately every 3 months until death. For patients without evidence of disease recurrence, including those patients who discontinued the study treatment, clinical and radiological assessments will be performed every 3 months the first year, every 4 months the second and third year, and every 6 months up to five years. After recurrence, survival information will be collected as stated above, approximately every 3 months until death or the study closes.

8.4. BIOMARKER TESTING AND BIOBANK

The key objective of the biomarker blood sampling is to investigate whether circulating tumor *KRAS* mutations in plasma are associated with DFS and OS and whether YKL-40 and IL-6 in plasma is correlated with DFS and OS. A biomarker blood sampling is mandatory.

Time points for additional blood samples for biomarker analyses prospectively collected at the same time that patients get routine blood tests as part of their treatment are:
• prior to the start of the first cycle of treatment
• before starting the second cycle
• at the time of CT-assessment

At before mentioned time points approximately 59 ml blood (for aliquots of whole blood in PAXgene tubes, serum, EDTA plasma and buffy coat) for biomarker analyses will be collected. The project blood samples will be stored at Herlev & Gentofte University Hospital BIOPAC biobank for up to 20 years after the end of the study. The model for the collection of blood samples, separation and freezing is according to the BIOPAC Study ("BIOmarkers in patients with Pancreatic Cancer). This biomarker study is approved by the Regional Ethics Committee (VEK ref. KA-20060113) and the Danish Data Protection Agency (j.nr. 2006-41-6848 and HGH-2015-027, I-Suite nr: 03960). We will use the same standard operating procedures (SOP) for the collection, handling and storage of blood samples.

The samples, as well as the attached identification list (access key) will be stored securely and separately. The samples will be kept after the completion of the study, but should be used only in the way patient has given his/her permission. The samples can be available to a new research project only after approval is obtained from the Regional Ethics Committee and the Danish Data Protection Agency.
The results of this study will be published in a scientific report, but without patient's identity to be obvious.

9. SELECTION AND WITHDRAWAL OF SUBJECTS

This phase 2 study will include 180 evaluable patients with histologically confirmed resected ductal pancreatic adenocarcinoma with macroscopic complete resection (R0 and R1).The patients will be recruited at Department of Oncology, Herlev& Gentofte Hospital, Denmark.

9.1. GENERAL ELIGIBILITY CRITERIA

Only patients that fulfill the inclusion criteria (9.3) and none of the exclusion criteria (9.4) are eligible for enrolment.

9.2. SCREENING AND ENROLMENT OF SUBJECTS

Potentially suitable patients will be screened by physicians or research nurses in order to find eligible participants. The screening procedure aims at confirming that the inclusion and exclusion criteria are met.

9.3. SUBJECT INCLUSION CRITERIA

Patients with:

1. Signed informed consent
2. Histologically confirmed resected ductal pancreatic adenocarcinoma with macroscopic complete resection (R0 and R1). Subjects with neuroendocrine (and mixed type) tumors are excluded
3. Subject should be able to start treatment no later than 12 weeks post-surgery
4. Male or non-pregnant, non-lactating females who are ≥18 years of age at the time of signing the informed consent form (ICF)
5. ECOG/WHO Performance Status (PS) 0-1
6. Females of child-bearing potential (defined as a sexually mature woman who (1) has not undergone hysterectomy [the surgical removal of the uterus] or bilateral oophorectomy [the surgical removal of both ovaries] or (2) has not been naturally postmenopausal for at least 24 consecutive months [ie, has had menses at any time during the preceding 24 consecutive months]) must:
   - Agree to the use of two physician-approved contraceptive methods (oral, injectable, or implantable hormonal contraceptive; tubal ligation; intra-uterine device; or vasectomized partner) while on study IP; and for 3 months following the last dose of IP
   - Has negative serum pregnancy test (β -hCG) result at screening
7. Male subjects:
   - Must practice true abstinence or partner (female of childbearing potential) shall agree to use an oral, injectable, or implantable hormonal contraceptive, tubal ligation or intra-uterine device during sexual contact, while patient participates
8. Understand and voluntarily sign an ICF prior to any study related assessments or procedures being conducted
9. Be able to adhere to the study visit schedule and other protocol requirements
10. Acceptable hematology parameters defined as:
    - Absolute neutrophil count (ANC) ≥ 1.5 x 10^9/L
    - Platelet count ≥ 100 x 10^9/L
    - Haemoglobin ≥ 5.6 mmol/L
11. Acceptable liver function defined as:
    - Serum bilirubin < 1.5 x upper limit of normal (ULN)
    - ASAT/ALAT < 2.5 x ULN
12. Calculated creatinine clearance ≥ 50 mL/min/ (eg, using the Cockroft-Gault formula)

9.4. SUBJECT EXCLUSION CRITERIA

Patients with:

1. Prior neo-adjuvant treatment, radiation therapy, or systemic therapy for pancreatic adenocarcinoma
2. Presence of or history of metastatic or locally recurrent pancreatic adenocarcinoma
3. Other malignancies, except adequately treated basal carcinoma or squamous cell carcinoma of the skin or *in situ* cervix carcinoma or incidental prostate cancer (T1a, Gleason score ≤ 6, PSA < 0.5 ng/ml), or any other tumor with a DFS of ≥ 5 years

4. History of serious or concurrent illness or uncontrolled medical disorder; any medical condition that might be aggravated by chemotherapy treatment or which could not be controlled; including, but not restricted to:
   - Active infection requiring antibiotics within 2 weeks before the study inclusion
   - Concurrent congestive heart failure NYHA (class III - IV)
   - Unstable angina pectoris, or myocardial infarction within 6 months and/or prior poorly controlled hypertension
   - History of interstitial lung disease, slowly progressive dyspnea and unproductive cough, sarcoidosis, silicosis, idiopathic pulmonary fibrosis, pulmonary hypersensitivity pneumonitis or multiple allergies
   - Concomitant use of immunosuppressive or myelosuppressive medications that would in the opinion of the investigator, increase the risk of serious neutropenic complications
5. Known or suspected allergy to the investigational agents or any agents given in association with this trial
6. Any psychological, familial, sociological, or geographical condition which does not permit protocol compliance and medical follow-up
7. Enrollment in any other clinical protocol or investigational study with an interventional agent or assessments that may interfere with study procedures
8. Any condition including the presence of laboratory abnormalities, which places the subject at unacceptable risk if he/she were to participate in the study
9. Any condition that confounds the ability to interpret data from the study
10. Unwillingness or inability to comply with study procedures
11. Current use of anticoagulation therapy such as heparins both unfractionated and low molecular weighted

9.5. CONTINUATION CRITERIA

Treatment according to the logarithm will not be disrupted unless any of the below listed criteria are valid (9.6).

9.6. WITHDRAWAL OF SUBJECTS

There may be unpredictable risks and harm by participating in the project. If new information is available on the efficacy, risks, side effects or complications of treatment while the project is ongoing, the subject will be notified about it in order to continue participation in the trial.

A participant will be withdrawn if:
- he or she withdraws his/hers informed consent at his/hers own request
- unacceptable toxicity not manageable by symptomatic therapy, dose delay or dose modification, including meeting stopping criteria for liver chemistry defined in chapter 9.3 and/or for hematologic and other nonhematologic toxicity (according to chapter 10)
- he or she does not comply with the instructions given by the study personnel
- unequivocal disease recurrence
- pregnancy
- investigator's discretion
- study is closed or terminated (according to chapter 22.4)

The reason and date of a withdrawal must be stated in the patient's CRF and in the medical records. Patients that have been withdrawn will continue to be tracked and assessed (unless the patient explicitly declines).

9.7. CONSIDERATIONS AFTER WITHDRAWAL

A participant that has been withdrawn (by any reason) cannot be re-included in the study. Their subject number cannot be reused.

10. TREATMENT OF SUBJECTS

10.1. TREATMENT ADMINISTRATION AND TREATMENT MODIFICATIONS

10.1.1. PRE-DEFINED DOSAGE SCHEDULE

In the investigational arm, T-ChOS™ and GEM/CAP are combined as follow:
- T-ChOS™ 600 mg daily p.o. in the morning
- GEM: 1000 mg/m² i.v. on day 1, day 8 and day 15 of every 28 day cycle for 6 cycles.
- CAP: 1660 mg/m²/day p.o. twice daily 21/28 day i. e. 24 weeks.

In the reference arm, placebo and GEM/CAP are combined as follow:
- Placebo: 600 mg daily p.o. in the morning
- GEM: 1000 mg/m² i.v. on day 1, day 8 and day 15 of every 28 day cycle for 6 cycles.
- CAP: 1660 mg/m²/day p.o. twice daily 21/28 day i. e. 24 weeks.

10.1.2. INDIVIDUAL DOSE MODIFICATIONS

Treatment will be modified in case of significant haematological and/or non-haematological toxicity. Dose adjustment and/or treatment delay are to be made according to the body system showing the greatest degree of toxicity. Any patient who requires a dose reduction will continue to receive a reduced dose for the remainder of the study. No dose re-escalation will be performed after dose reduction.

Dose adjustments at the start of a subsequent cycle should be based on nadir haematological counts or maximum non-haematological toxicity from the preceding cycle of therapy. Treatment may be delayed to allow sufficient time for recovery. Upon recovery, patients should be retreated using the guidelines in the following tables. If the study treatment cannot be administered after a 2 week delay (cycle duration > 6 weeks) because of any toxicity, decision on continuation of treatment should be thoroughly considered.

- For both treatment arms, to start a new treatment cycle, ANC $\geq 1.5 \times 10^9$ and platelets $\geq 100 \times 10^9$ are required. For non-haematological toxicity, all toxicity, except alopecia, fatigue, nausea or vomiting, should have recovered to CTCAE grade ≤ 1 or baseline. The onset of a new treatment cycle should be withheld until the criteria's for starting a new cycle are met. If the criteria are not met within 14 days the decision on continuation of treatment should be thoroughly considered (see chapter 10.1.2.5).

- For both treatment arms the dose of T-ChOS™ on day 1, gemcitabine and capecitabine on day 1, 8 and 15 should be adjusted according to the following table:

Table 3: Dose Modifications

Dose Level	T-ChOS™/Placebo (mg)[a]	GEM (mg/m^2)[a]	CAP (mg/m2)[a]
Study Dose	600	1000	1660
-1	600	800	1300
-2[b]	600	600	900

a) Dose reductions may or may not be concomitant, refer to Table 4 and 5. If the dose is withheld due to hematologic toxicity on Day 15, resume at the next lower dose level when the subject has adequate ANC and platelet counts to begin Day 1 of the next cycle. Refer to Table 4 and 5 for specific recommendations regarding dose modifications for Day 1.
b) A maximum of 2 dose level reductions are allowed.

10.1.2.1. Dose modification for both Investigational and Control arm: T-ChOS™/placebo with GEM/CAP

In the event dose modifications are required at the start of a cycle or within a cycle due to hematologic toxicities of neutropenia and/or thrombocytopenia, doses of T-ChOS™/placebo, GEM og CAP may be adjusted as detailed in Table 4. WBC growth factor may be given according to institutional guidelines for the treatment of neutropenic fever or infections associated with neutropenia and for the prevention of febrile neutropenia in subjects with an ANC < 500 cells/mm^3. Subjects' not experiencing resolution of neutropenia within 14 days, despite uninterrupted WBC growth factor treatment, will be considered for discontinuation of study treatment. In addition, WBC growth factors may be administered as supportive therapy to recover ANC adequately such that dosing levels may be maintained. If a dose reduction was required due to neutropenia or other reason no dose re-escalation will be permitted for the duration of the study.

The doses of T-ChOS™/placebo and gemcitabine in the next treatment cycle should be adjusted according to the following table:

Table 4: Dose Recommendation and Modifications for Neutropenia and/or Thrombocytopenia at the Start of a Cycle or Within a Cycle

Cycle Day	ANC (cells/mm^3)		Platelet count (cell/mm^3)	T-ChOS™/placebo Dose	GEM Dose	CAP Dose
Day 1	≥ 1500	AND	≥ 100,000	Treat on time at current dose levels		
	< 1500	OR	< 100,000	Treat on time at current dose levels*	Delay doses until recovery	
Day 8	≥ 1000	AND	≥ 75,000	Treat on time at current dose levels		
	≥ 500 but < 1000	OR	≥ 50,000 but <75,000	Treat on time at current dose levels*	Reduce doses 1 dose level	
	< 500	OR	< 50,000	Treat on time at current dose levels*	Withhold doses	
**Day 15: IF Day 8 gemcitabine doses were given without modification:**						
Day 15	≥ 1000	AND	≥ 75,000	Treat on time at current dose levels		

	≥ 500 but < 1000	OR	≥ 50,000 but < 75,000	Treat on time at current dose levels*	Reduce doses 1 dose level from Day 8; consider following with WBC growth factors for support**
	< 500	OR	< 50,000	Treat on time at current dose levels*	Withhold doses
**Day 15: IF Day 8 gemcitabine doses were reduced:**					
Day 15	≥ 1000	AND	≥ 75,000	Treat with same doses as Day 8; consider following with WBC growth factors for support**	
	≥ 500 but < 1000	OR	≥ 50,000 but < 75,000	Treat on time at current dose levels*	Reduce doses 1 dose level from Day 8; consider following with WBC growth factors for support**
	< 500	OR	< 50,000	Treat on time at current dose levels*	Withhold doses
**Day 15: IF Day 8 gemcitabine doses were withheld:**					
Day 15	≥ 1000	AND	≥ 75,000	Option A: Maintain dose level from Day 1 and follow with WBC growth factors for support** OR Option B: Reduce doses 1 dose levels from Day 1	
	≥ 500 but < 1000	OR	≥ 50,000 but < 75,000	Treat on time at current dose levels*	Option A: Reduce 1 dose level from Day 1 and follow with WBC growth factors for support** OR Option B: Reduce doses 2 dose levels from Day 1
	< 500	OR	< 50,000	Treat on time at current dose levels*	Withhold doses

Abbreviations: ANC = absolute neutrophil count; WBC = white blood cell.

* At the discretion of investigator

**The use of WBC growth factors is only applicable if the dose limiting hematologic toxicity was limited to neutropenia or febrile neutropenia.

Dose modifications for other adverse drug reactions are provided in Table 5.

Table 5: Dose Modifications for Other Adverse Drug Reaction

Adverse Drug Reaction	T-ChOS™/placebo (mg)[a]	GEM (mg/m^2)[a]	CAP (mg/m^2)[a]
Febrile neutropenia[a]: Grade 3 or 4	Treat with same dose	Withhold doses until fever resolves and ANC is ≥ 1500; resume at next lower dose level[b]	
Diarrhea: Grade 3 or 4	Treat with same dose	Treat with same dose	Withhold dose until improvement to ≤ Grade 1; resume at next lower dose level[b]
Cutaneous toxicity: Grade 2 or 3	Treat with same dose	Reduce doses to next lower dose level[b]; discontinue treatment if ADR persists	
For all other non-hematologic toxicities (except nausea, vomiting, alopecia and pulmonary embolism[c]) of ≥ Grade 3	Treat with same dose	Withhold doses until improvement to ≤ Grade 1; resume at next lower dose level[b]	

Abbreviations: ADR, adverse drug reaction; ANC = absolute neutrophil count

[a] White blood cell growth factor may be given according to institutional guidelines for the treatment of neutropenic fever or infections associated with neutropenia and for the prevention of febrile neutropenia in subjects with an ANC of <500 cells/mm^3

[b] See Table 3 for dose level reductions. Dose reductions may or may not be concomitant, i.e. diarrhea or PPE due to CAP.

[c]See Section 10.1.2.3.

10.1.2.2. Administration of IP to Subjects with Abnormal Hepatic Function

IP should only be administered if hepatic function is within the parameters established in the eligibility criteria. Mild hepatic toxicity may occur but severe liver injury is quite uncommon. Thus, hepatic dysfunction that occurs while the subject is on study should prompt an evaluation to determine the cause, including the possibility of metastatic disease and hepatotoxicity from concurrent medications, alcohol use, or other factors.

10.1.2.3. Pulmonary Embolism and Deep-vein Thrombosis

In the event of a pulmonary embolism or deep-vein thrombosis, treatment should be interrupted and subjects permanently discontinued from further study drug treatment.

10.1.2.4. Interstitial Pneumonitis

1. While participating in this study, subjects should be carefully monitored to prevent or minimize the occurrence of interstitial pneumonitis. Careful prestudy screening with continuous on-study monitoring for signs and symptoms is required. Should a subject develop symptoms of pneumonitis during this study, the timely initiation of appropriate management is required.
2. Radiographic evaluation with chest x-rays and CT scans (normal or high resolution) may be indicated to evaluate for infiltrates, ground-glass opacities, or honeycombing patterns. Pulse oximetry and pulmonary function tests can show respiratory and ventilation compromise.
3. Infections should be ruled out with routine immunological/ microbiological methods. Transbronchial lung biopsy is not recommended, given its limited value and risk of pneumothorax and hemorrhage, and should be reserved for cases with unclear etiology.
4. Treatment should be interrupted upon diagnosis of interstitial pneumonitis and subjects permanently discontinued from further study drug treatment. Patients should be managed according to clinical routine.

10.1.3. DOSE DELAY

If any toxicity, except alopecia, fatigue, nausea or vomiting, has not decreased to CTCAE ≤ 1 or baseline within 14 days, or criteria to start a new treatment cycle has not been reached within 14 days, treatment will be considered for discontinuation by sponsor assessment.

10.2. EXPECTED, MANAGEABLE TREATMENT RELATED TOXICITY

In the investigational arm of this study, two registered compounds (gemcitabine and capecitabine) will be used in combination with T-ChOS^{TM}. The side effect profile of GEM given in monotherapy and in combination with CAP is well known and well characterized. In the present protocol, the below listed side effects are considered expected and should be managed according to clinical routine. T-ChOS^{TM} has been tested in a phase II study for osteoarthritis and over 100 volunteers have ingested T-ChOS on a daily basis for various periods of time, ranging from 3 months to 11 years. No adverse reactions have been reported by volunteers, patients in phase 2 study or in PK studies performed in human subjects. Animal safety studies performed by Genis show that only after intravenously injecting a super dose of T-ChOS^{TM}, which reaches 1000-fold higher plasma concentration than the daily recommended dose, for 7 consecutive days minor skin

irritation appears in the paw region. This inflammation subsides once frequency of intravenous injections of this super dose is reduced to every other day. All these data support the exceptional safety and biocompatibility profile of chitin related products as reported in the literature.

In the previous phase II study for osteoarthritis and observation of more than 100 volunteers, ingesting T-ChOS™, the following events were reported. None of these events were related to T-ChOS according to invetsigators.

Mild	Anorexia, nausea, increased sexual appetite, dyspnea, heartburn, early satiety
Moderate	Knees paresthesia, increased appetite, dry mouth, polydipsia
Severe	Increased pain in knee, rash (abdomen and chest), pruritus, dizziness, general tremor

10.2.1. LIST OF COMMON AND EXPECTED TREATMENT RELATED TOXICITY

Myelotoxicity	Fatigue
Infections	Fluid retention / edema
Anorexia	Alopecia
Nausea	Rash
Vomiting	Dyspnoea
Diarrhea	Tremor
Stomatitis	Dizziness
Hand-and-Foot Syndrome	Pyrosis
Abdominal Pain	Polydipsia
Constipation	Xerostomia
Dyspepsia	Transaminase elevations
Myalgia including artralgia	Fever

10.3. CONCOMITANT THERAPY

Concomitant therapy is defined as any drug taken by the patient up to 30 days prior to the start of the study treatment or at any time during the study and up to 30 days after ending the study treatment. All treatments that are taken accordingly are regarded as concomitant treatment and must be recorded in the CRF.
Patients are requested to inform study personnel about any changes in concomitant treatments.

10.3.1. RECOMMENDED CONCOMITANT TREATMENTS

Over the course of this study, additional medications may be required to manage aspects as consequences of operation and side effects from study treatments. Supportive care, including but not limited to antiemetic medications, may be administered at the discretion of the investigator.

WBC growth factors may be administered at the discretion of the investigator, consistent with institutional guidelines (refer to Section 10.1.2.1 for details). WBC growth factors should be used only in patients who have demonstrated prior events of neutropenia; primary prophylaxis with WBC growth factors is not permitted. Erythropoietin may be administered at the discretion of the investigator, consistent with institutional guidelines.

For information regarding other drugs that may interact with gemcitabine and affect their metabolism, pharmacokinetics, or excretion, please see the gemcitabine package inserts (refer to Prescribing Information).

10.3.2. PROHIBITED AND RESTRICTED THERAPIES DURING THE STUDY

Radiotherapy increases the effect of gemcitabine. Radiotherapy should not be given 7days before or after treatment with gemcitabine.

Concomitant use of anticoagulation therapy such as heparins both unfractionated and low molecular weighted is not allowed due to chitin's binding ability to the heparin-binding domain. As well as concomitant use of coumarin-derivative anticoagulants such as warfarin and phenprocoumon is contraindicated due to an inhibition of cytochrome P450 2C9 by capecitabine and/or its metabolites.

Care should be exercised when capecitabine is coadministered with CYP2C9 substrates.
Concomitant use of yellow fever vaccine is contraindicated due to the risk of fatal generalized vaccinal disease. Concomitant use of live attenuated vaccines is not recommended due to the risk of systemic, possible fatal disease.

Administration of other chemotherapy, immunotherapy, or antitumor therapy during the study is not allowed until documented disease recurrence.

10.3.3. SUPPORTIVE AUTHORIZED TREATMENTS

Patients should receive full supportive treatment including steroids as antiemetic, opiates in case of pain, bisphosphonate therapy, antibiotics, antidiarrhoeals, and transfusion of blood products, when appropriate.

10.4. COMPLIANCE WITH THE TREATMENT

The nurse responsible for drug administration to the patient will check the patient's name and identity (the patient will need to state his/hers date of birth and social security number (if applicable)). The following information must be recorded in the patient record: drug name, date and time of administration (start, end). Any interruption must be stated in the patient record. If any extra antiemetic or fluid was administered this must also be recorded.

10.5. PACKAGING AND LABELLING OF INVESTIGATIONAL PRODUCTS

Commercially available T-ChOS, placebo, capecitabine and gemcitabine vials from the local pharmacy will be administered in this study. The content of the labelling is in accordance with requirements set by the regulatory authorities. T-ChOS, T-ChOS-placebo must be dispensed or administered only to subjects enrolled in the study and in accordance with the protocol.

10.6. BLINDING

Study treatment will be double-blinded. Genis, KFE site personnel (including the investigator) and the subject will not know the treatment assignment. Every effort must be made to maintain the blind until all analyses have been performed. The investigator or treating physician may unblind a subject's treatment assignment only in the case of an emergency, when knowledge of the study treatment is essential for the appropriate clinical management or welfare of the subject. Whenever possible, the investigator must first discuss options with the monitor or appropriate Genis study personnel before unblinding the subject's

treatment assignment. If this is impractical, the investigator must notify Genis as soon as possible, but without revealing the treatment assignment of the unblinded subject, unless that information is important for the safety of subjects currently in the study. The date and reason for the unblinding must be recorded in the eCRF. A subject should remain in the study for survival follow-up even if the treatment code is unblinded.
If the SAE requires that an expedited regulatory report be sent to one or more regulatory agencies, a copy of the report, identifying the subject's treatment assignment, may be sent to clinical investigators in accordance with local regulations.

11. ASSESMENT OF EFFICACY

11.1. TUMOUR ASSESMENT

CT scan of the thorax and abdomen is required at baseline (within 28 days before first study drug administration). Tumor recurrence will be evaluated at baseline and every 12 weeks/3nd cycle. No confirmation of recurrence is required if any objective response is documented. If the patient stops treatment due to unacceptable toxicity or any other reason other than tumor recurrence, radiological evaluation will continue every 3 months the first year, every 4 months the second and third year, and every 6 months up to five years or until radiological relapse. Tumor lesions will be categorized as measurable and/or non-measurable lesions using the RECIST1.1 criteria defined as:

- Measurable disease: lesions that can be measured in at least one dimension and which have not been previously irradiated. Longest diameter (LD) ≥ 20 mm with conventional techniques or >10 mm with spiral CT scan or MRI.
- Non-measurable disease: lesions which have not been previously irradiated. Longest diameter <20 mm with conventional techniques or <10 mm with spiral CT scan or MRI, or truly non measurable lesions including bone lesions, ascites, pleural/pericardial effusion, and lymphangitis cutis/pulmonitis.

12. ASSESSMENT OF SAFETY

The below listed tests (12.1-12.5) will be performed prior to and on specified days for the duration of study.
Gemcitabine has been explored in a Phase III study for treatment of advanced pancreatic cancer. The trial showed a good toxicity tolerance profile.

12.1. LABORATORY ASSESSMENTS

Laboratory tests include:

- Haematology: Hb, Platelets, WBC including ANC
- Electrolytes: P-Na, P-K, P-Ca, P-Alb
- Hepatic function: P-ASAT, P-ALAT, P-bilirubin, P-ALP, P-LDH
- Renal function: P-creatinine, e-GFR
- CRP
- CA19-9

12.2. BLOOD PRESSURE AND HEART RATE

It is compulsory to measure and record (in the CRF) blood pressure and heart rate at each visit.

12.3. ELECTROCARDIOGRAM (ECG)

At baseline a 12-lead electrocardiogram is required.

12.4. ADVERSE EVENTS (AE)

An adverse event (AE) is any untoward medical occurrence in a patient or clinical investigation subject administered a pharmaceutical product that does not necessarily have a causal relationship with the treatment. An AE can be any unfavorable and unintended sign (including a laboratory finding), symptom or disease temporally associated with the use of a medicinal (investigational) product, whether or not related to the medicinal (investigational) product.

12.4.1. METHODS FOR ELICITING, RECORDING AND FOLLOW-UP OF ADVERSE EVENTS

AEs should be recorded from signing of the ICF. AEs occurring before the first dose of study product will be considered a non-treatment emergent (baseline) AE and should be recorded on the Medical History eCRF page. At each AE assessment, patients should be interviewed to elicit potential adverse reactions from the patient. The occurrence of an AE will be based on clinically significant changes, in the opinion of the Investigator, in the patient's physical examination, laboratory results, and/or signs and symptoms.
Only Grade 3 and 4 laboratory abnormalities that are considered by the investigator to be clinically significant need to be recorded as AEs. Generally, low grade laboratory abnormalities or those that do not require an intervention, will not be considered as clinically significant.
Expected progression of the patient's disease and/or expected progression of signs and symptoms of the disease should not be reported as an AE. Any events that are unequivocally due to disease progression (according to RECIST 1.1) must not be reported as an AE.
Surgical procedures themselves are not AEs; they are therapeutic measures for conditions that require surgery. The condition for which the surgery is required is an AE, if it occurs or is detected after the administration of the IMP. Planned surgical measures permitted by the clinical study protocol and the conditions leading to these measures are not AEs, if the condition was known before the start of period of observation. In the latter case the condition should be reported as medical history.

All AEs regardless of causal relationship are to be recorded on the Adverse Event eCRF page. Information to be collected includes event description, date and time of onset, Investigator's assessment of severity, relationship to study product, time of resolution/stabilization and final outcome of the event.
AEs will be followed until resolution or stabilization while the patient remains on-study. Once the patient is removed from study, events thought to be related to the study medication will be followed until resolution or stabilization, unless, in the Investigator's opinion the event is unlikely to resolve due to the patient's underlying disease, or until the patient starts a new treatment regimen or the patient is lost to follow-up.

12.4.2. SEVERITY OF ADVERSE EVENTS

The Investigator must determine the severity of any AEs according to the National Cancer Institute (NCI) Common Terminology Criteria for Adverse Events (CTCAE) Version 4.0 (see http://ctep.info.nih.gov). Those AEs not covered by these criteria will be graded as follows:
Grade 1. Mild; asymptomatic or mild symptoms; clinical or diagnostic observations only; intervention not indicated.

Grade 2. Moderate; minimal, local or noninvasive intervention indicated; age limiting appropriate instrumental activities of daily living (ADL)*.
Grade 3. Severe or medically significant but not immediately life-threatening; hospitalization or prolongation of hospitalization indicated; disabling; limiting self-care ADL**.
Grade 4. Life-threatening consequences; urgent intervention indicated.
Grade 5. Death related to AE.
*Instrumental ADL refer to preparing meals, shopping for groceries or clothes, using the telephone, managing money, etc.
**Self-care ADL refer to bathing, dressing and undressing, feeding self, using the toilet, taking medications, and not bedridden.

12.4.3. ATTRIBUTION DEFINITIONS

The Investigator is obligated to assess the relationship between the study product and the occurrence of each AE/SAE that occurs during the study by answering the causality question "Is there a reasonable possibility that the AE may have been caused by the study product?"
An AE is considered to be associated with the use of the study product if the attribution is determined as likely. Attribution of AEs will be recorded on the eCRF page as:
• Likely: There is a reasonable possibility that the AE is related to the study treatment (i.e., related).
• Unlikely: There is not a reasonable possibility that the AE is related to the study treatment (i.e., not related).

12.4.4. FINAL OUTCOME OF EVENT

The following terms are used when assessing the final outcome of an AE:
• Recovered/resolved – the patient has fully recovered or the condition has returned to the level observed when the patient was screened for the study.
• Recovering/resolving – the condition is improving and the patient is expected to recover from the event.
• Recovered/resolved with sequelae – the patient has recovered from the condition, but with lasting effect due to disease, injury, treatment or procedure.
• Not recovered/not resolved – the condition of the patient has not improved and the symptoms are unchanged, or the outcome is not known.
• Fatal – this term is only applicable if the subject died from a condition related to the reported AE. An AE with fatal outcome must be reported as an SAE.
• Unknown – this term is only applicable if the patient is lost to follow-up.

12.4.5. DEFINITION OF AN UNEXPECTED ADVERSE EVENT

An unexpected AE is defined as any adverse product experience, the specificity or severity of which is not consistent with the current T-CHOS Investigator's Brochure.
Unexpected, as used in this definition, refers to an adverse experience that has not been previously observed (e.g., included in the T-CHOS Investigator's Brochure) rather than from the perspective of such experience not being anticipated from the pharmacological properties of the pharmaceutical product.

12.4.6. DEFINITION OF SERIOUS ADVERSE EVENTS (SAE)

A serious adverse event (SAE) includes, but is not limited to an event which:

- results in death
- is life-threatening
- results in persistent or significant disability
- requires acute or unplanned hospitalization or prolongation of existing hospitalization
- is a congenital anomaly or birth defect

- medically important event (in which the investigators finds or suggests a significant hazard)

Exceptions:
- In this trial, events and symptoms due to progression of the cancer shall not be reported as SAE.
- Hospitalization due to study procedures or due to cancer morbidity shall not be reported as SAE.
- Hospitalization due to below mentioned expected treatment related toxicity shall not be reported as SAE:

Myelotoxicity	Fatigue
Infections	Fluid retention / edema
Anorexia	Alopecia
Nausea	Rash
Vomiting	Dyspnoea
Diarrhea	Tremor
Stomatitis	Dizziness
Hand-and-Foot Syndrome	Pyrosis
Abdominal Pain	Polydipsia
Constipation	Xerostomia
Dyspepsia	Transaminase elevations
Myalgia including artralgia	Fever

Any serious event up to 30 days after ending study treatment must be reported.

12.5. ADVERSE REACTIONS (AR)

12.5.1. DEFINITION OF ADVERSE REACTION (AR)

An Adverse Reaction of an investigational medicinal product is defined as any noxious and unintended response to a medicinal product related to any dose administered.

All adverse events judged by either the reporting investigator or the sponsor as having a reasonable causal relationship to a medicinal product qualify as adverse reactions. The expression reasonable causal relationship means to convey in general that there is evidence or argument to suggest a causal relationship.

12.5.2. UNEXPECTED ARVERSE REACTION (UAR)

An Adverse Reaction, the nature or severity of which is not consistent with the applicable product information (e.g. investigator's brochure for an unauthorised investigational product or summary of product characteristics for an authorised product).

When the outcome of the adverse reaction is not consistent with the applicable product information this adverse reaction should be considered as unexpected.

12.5.3. SERIOUS ADVERSE REACTION (SAR)

Serious Adverse Reaction is defined as any SAE which is considered related to the protocol treatment.

12.5.4. DEFINITION OF SUSPECTED UNEXPECTED SERIOUS ADVERSE REACTION (SUSAR)

Suspected unexpected serious adverse reaction (SUSAR) is any serious event that is suspected to be connected to the study treatment and not described in the Summary of Product Characteristics (SPC). The sponsor will decide if the SADR (Serious Adverse Drug Reaction) is a SUSAR or not.

12.6. REPORTING OF SERIOUS ADVERSE EVENT (SAE) AND SUSPECTED UNEXPECTED SERIOUS ADVERSE REACTION (SUSAR)

All serious adverse events, as listed above and suspected unexpected serious adverse reaction must be documented by the investigator immediately as he or she is notified of the event.
The sponsor must report to the Regulatory Authorities, all SUSARs that are lethal or life threatening within 7 days and any relevant complementary information within another 8 days. All other SUSARs shall be reported within 15 days. Serious adverse reactions related to T-ChoS as assessed by investigator will be reported to Danish Veterinary and Food Administration.

The sponsor will perform all safety reports pursuant to applicable laws and regulations as set by the Regulatory Authorities and Ethics Committees.

12.7. FOLLOW-UP OF SERIOUS ADVERSE EVENTS

All serious adverse events must be followed-up and recorded. The serious adverse events must be followed-up until resolution or stabilization.

12.8. ANNUAL SAFETY REPORT

Once a year throughout the clinical trial, the sponsor will provide an updated safety report highlighting SARs and SUSARs to the Regulatory Authorities and Ethics Committee.

12.9. TRIAL SAFETY COMMITTEE

The sponsor will designate a Trial Safety Committee to be eligible to stop trial for safety reasons.

13. ASSESSMENT OF QUALITY OF LIFE

To assess Quality of Life the EORTC Quality of Life Questionnaire C30 (QLQ-C30) Version 3.0 will be used. The EORTC QLQ questionnaire was designed to be cancer specific, multidimensional in structure, appropriate for self-administration, and applicable across a range of cultural settings. The EORTC QLQ-C30 version 3.0 is the most recent version (http://groups.eortc.be/qol/qlq-c30-development). See Appendix 26.6.
The QLQ-C30 should be filled in and registered prior to treatment start (screening visit), after every third cycle and at the follow-up visit.

14. STATISTICS AND DATA MANAGEMENT

14.1. DATA MANAGEMENT

14.1.1. DATA ENTRY AND DATA VALIDATION

Data collected in the CRFs will be entered into a data base. Data will be extracted from the data base for statistical analysis. Random samples might be inspected by the Danish Data Protection Agency for whether the processing of data is carried out in accordance with the Act on Processing of Personal Data.

14.1.2. CODING

Adverse events on this protocol are graded according to the National Cancer Institute Common Toxicity Criteria version 4.0.

14.2. STATISTICAL ANALYSIS

The study is designed as a phase II trial and therefore, for a statistically significant difference in the primary endpoint favoring the experimental arm, the superiority of the experimental combination will be interpreted as a signal of effect. A definitive phase III trial with survival endpoint will be necessary to confirm the results.

14.2.1. OBJECTIVES AND ENDPOINTS

The study is designed as a randomized, double blind phase II trial, with the primary endpoint DFS. The Kaplan-Meier estimator will be used to estimate DFS and differences in DFS will be tested using the log-rank test. Treatment effect adjusted for any differences in two groups will be estimated using Cox proportional hazards regression. The median DFS after adjuvant treatment with conventional treatment is approximately 14 month. If the true hazard ratio (relative risk) of control subjects relative to the experimental subjects is 0.6 we will need to study 90 patients in the investigational arm and 90 patients in the reference arm to be able to reject the null hypothesis at a 10% significance level that the experimental and control survival curves are equal with probability (power) of 90%. With an annual accrual rate of 70 patients per year during three years and extra two years of follow-up the study is expected to generate the necessary number of events within a five year period.
DFS is defined as survival from the date of randomization until date of recurrence.

14.2.2. ANALYSES

The primary endpoint DFS will be analyzed using survival techniques. Survival curves will be estimated using the method of Kaplan-Meier, and differences in survival times will be tested using the log-rank test. Proportional hazards regression will be used to estimate the effect of treatment on DFS. Both the unadjusted effect – a model with only treatment included – as well as the adjusted effect – a model with the stratification factors as well as treatment included - will be estimated. Results of the analyses will be presented as hazard ratios (HRs) together with confidence intervals. If the upper limit of a one-sided 90% confidence interval for the HR (experimental arm versus control arm) in the analysis of DFS is less than 1, the experimental treatment is to be considered for further investigation in a phase III trial with overall survival as primary endpoint.

OS and differences in the OS rate will be analyzed using the same techniques as DFS.

The effect of treatment on QoL will be evaluated using linear mixed models.

Other secondary and exploratory endpoints will be evaluated by using descriptive methods and statistics. All patients that have received at least one treatment cycle will be included in the safety and QoL analyses.
All analyses of primary and secondary endpoints will be based on the intention-to-treat principle.
The statistical analyses will be performed by the chief investigators, the statisticians and affiliated members.

14.3. RANDOMIZATION AND STRATIFICATION OF SUBJECTS

Patients should be randomized after written informed consent is obtained and all screening assessments have been performed. Centralized randomization will be performed by the Clinical Trial Unit/Department of Oncology, Herlev University Hospital, Denmark.
Randomization will based on permuted block technique and stratified on the following factors:

- Nodal Status: lymph node (LN)+ versus LN-
- Resection Status: R 0 (tumor-free margin) versus R1 (microscopically positive margin)

15. TRANSLATIONAL ANALYSES

In addition to the major objectives of the trial, outlined in section 7.1 and 7.2, an exploratory translational part of the trial aim to analyze presumptive biomarkers and therapy induced changes of these markers that may have predictive and prognostic value and deserve further evaluation and validation in larger phase III trials. At Herlev Hospital translational research will be performed for all patients participate in the study. Consecutive sampling of plasma (59 ml) will be collected at baseline, and every 4th weeks (i.e. prior every treatment cycle).

This biomaterial will be used for analyses such as:

a) Plasma YKL-40 and IL-6 and its relation with DFS and OS.
b) Circulating tumor DNA (ctDNA) *KRAS* mutations in plasmain relation with DFS and OS.

YKL-40 concentrations will be determined in duplicates in EDTA plasma samples by a commercial enzyme-linked immunosorbent assay (ELISA) (Micro Vue YKL-40, Qudel, San Diego, CA, USA) according to the manufacturer's instructions. The detection limit 10 ng/l, and intra- and inter-assay CVs were ≤ 5% and ≤ 6%.
IL-6 concentrations will be determined in duplicates in EDTA plasma samples by a commercial enzyme-linked immunosorbent assay (ELISA) (Catalogue number HS600, R&D Systems, Abingdon, Oxon, UK) according to the manufacturer's instructions. The detection limit 0.01 ng/l, and intra- and inter-assay CVs were ≤ 8% and ≤ 11%. Correlations between IL-6 and clinical outcome, as well as normalization of C-reactive protein (CRP), improvement of fatigue, development of cancer cachexia will be explored.
Circulating DNA *KRAS* mutation load will be determined in EDTA plasma by a commercial Trovagene Precision Cancer Monitoring (PCM) *KRAS* platform test (single copy detection: 0.0055% analytical sensitivity) using a proprietary method for extracting circulating tumor DNA (ctDNA) and a PCR assay to amplify small DNA fragments while enriching for mutant alleles. DNA fragments will be detected by next-generation sequencing (NGS). Correlations between circulating DNA *KRAS* mutation load and clinical outcome will be explored.

16. DIRECT ACCESS TO SOURCE DATA/DOCUMENTS

The Investigator will permit study-related monitoring, audits, IEC review and regulatory inspection(s), providing direct access to source data/hospital records. The Investigator verifies that each subject has consented in writing to direct access to the original source data/hospital records by the use of written patient information and signed Informed Consent.
The selected data recorded in the CRFs will be controlled for consistency with the source data/hospital records during the monitoring (source data verification). Any discrepancies of data will be documented and explained in the monitoring reports.

17. QUALITY CONTROL AND QUALITY ASSURANCE

17.1. SOURCE DATA

Source data is defined as all information in original electronic records of clinical documentation and certified copies of original records of clinical findings, observations, or other activities in a clinical trial necessary for the reconstruction and evaluation of the trial. Source data are contained in source documents (original electronic records or certified copies).

Source documents include documents, data, and records such as:

- Signed Informed Consent
- Eligibility Criteria
- Demographic Details (OPUS, EPIC)
- Hospital electronic records, clinical and office charts (OPUS, EPIC)
- Pathology records (Patobank)
- Diagnostic reports (OPUS, EPIC)
- Laboratory results (OPUS, EPIC)
- Subjects' diaries or evaluation checklists
- Concomitant Medications (OPUS, EPIC)
- ECG (MUSE-Web, EPIC)
- Documentation of study drugs (EPIC, HOBS, Administration)
- CTC worksheet
- Records kept at the pharmacy, at the laboratories and at medico-technical departments involved in the clinical trial
- BIOPAC Database

The patient must have agreed to allow their medical records to be examined by authorized personnel of the sponsor (or thereof affiliated).

17.2. STUDY MONITORING

The Copenhagen GCP unit is appointed for study monitoring. The Clinical Trial Unit at the Herlev & Gentofte University Hospital of Copenhagen will instruct pre-study investigators and personnel about the protocol, trial procedures, reporting of outcome and any serious adverse events. The pre-study controls aim at confirming that the site complies with the requirements as specified in the protocol and GCP guidelines.

18. ETHICS

18.1. INDEPENDENT ETHICS COMMITTEE

Approval of the study protocol (including any protocol amendment, patient information and Informed Consent forms) must be obtained from the regional Ethics Committee (EC) before any subject enters into the study. The written approval from the EC should be dated and have an attached list of those persons (with name and positions) present at the EC meeting.
It is the responsibility of the Investigator to report all SARs and SUSARs to the IEC. Further the Investigator will report when the study is completed or if the study for any reason stops prematurely.

18.2. ETHICAL CONDUCT OF THE STUDY

This study will be conducted in accordance with the protocol, ICH-GCP and the ethical principles of the Declaration of Helsinki as adopted by the 18th World Medical Assembly in Helsinki, Finland, in 1964 and subsequent versions. The data will not identify any subject taking part in the study, in accordance with the EU Data Protection Directive (95/46/EU) and the Law on Personal Data (Persondataloven nr. 429 of May 2000). All patients will be informed both orally and in writing form about the purpose of the study, and after consideration, patients must give verbal and written consent before enrollment. It is emphasized that participating is on a voluntary basis and the patient at any time may cancel participation in the trial. This will not affect the patient's treatment or relationship with the department in general.

All newly referred individuals with resected PC who meet inclusion and exclusion criteria are likely to be included. The standard adjuvant treatment for patients after operation for pancreatic cancer is 6 months of gemcitabine and capecitabine. The OS is about 28 months. New more effective regimens in terms of inhibition of cancer relapse are needed.

The combination of gemcitabine and capecitabine with T-ChOS™ has not been evaluated in the adjuvant treatment of patients operated for PC. High levels of YKL-40 in patients with cancer, including PC, are associated with short OS (25, 29, 30). Thus, we believe that blockade of YKL-40-regulated signaling pathways represents a promising approach in combination with gemcitabine and capecitabine.

Gemcitabine is well established, approved treatment for patients with PC in both palliative and adjuvant purposes. Gemcitabine is used routinely outside the trial on the same indications and same dose as in the trial, and is considered as standard adjuvant treatment for patients after operation. The recommended dose of gemcitabine 1000 $mg/m^2$was established. The most common side effects for gemcitabine: neutropenia, infections, fatigue, nausea, vomiting, diarrhea, muscle and joint pain including arthralgia, changes in liver function tests, fluid retention / edema, dryness and mouth irritation. Capecitabine in combination with gemcitabine has been compared with gemcitabine in the ESPAC-4 trial for resected pancreatic cancer (9). Combination improved survival compared to monotherapy with gemcitabine. The recommended dose of capecitabine is 1660 mg/m2/day. Only slightly more toxicity in the combination arm versus monotherapy gemcitabine was observed, 26 % and 25 %, respectively. Common side effects of capecitabine are skin toxicity, especially hand/foot syndrome, diarrhea and stomatitis. T-ChOS™ has been ingested by over 100 volunteers with various inflammatory disorders, has been tested in a phase II study with established doses of 700 mg p.o. daily and has been tested in a PK study with a single 1400 mg dose. No serious adverse effects that can be linked to T-ChOS™ have been reported to Genis in any of these studies and no complaints have been filed by regular users of T-ChOS™ in the last 11 years.

Trial Safety Committee will also be eligible to stop trial for safety reasons at any time during the study. The subjects will be fully informed about the risk and investigators will be aware of the occurrence of any side effects. Patients will be asked systematically about these side effects at each visit, like as the study participants will be instructed to contact the investigators if there occurs troublesome side effects between visits.

In spite which treatment arm the patient is treated according to, the chemotherapeutic drug in each arm is well used in clinical practice, well tolerated and with known tumor efficacy in pancreatic carcinoma. The combination in the investigational arm, T-ChOS™ with GEM/CAP, is not earlier studied in pancreatic carcinoma but is promising and of great interest. The results will have implications for future patients if investigational combination proves to be more effective than standard treatment.

The patients will be monitored closely and any adverse events will be followed up. Whenever there is a medication available to prevent or alleviate a symptom, it will be offered to the patient. Unacceptable side

effects will always be cause for immediate termination of the study. There may be unpredictable risks and harm by participating in the project. If new information is available on the efficacy, risks, side effects or complications of treatment while the project is ongoing, the subject will be notified about it in order to continue participation in the trial.

Patients will have taken the extra blood tests while they are still undergoing routine blood tests and thus should not be pricked more often. At the same time a patient undergoes routine blood samples (18 ml) there will be taken additional blood (59 mL) for determination of different biomarkers, which does not pose patients at risk. Blood sampling may be associated with some discomfort or short-term pain (prick) and one can then get bruises (hematoma). The risk of infection associated with the blood sampling can be considered to be quite minimal.

In connection with CT scans patients will be exposed to a certain dose of X-rays. Radiation dose for each X-ray examination is about 10 mSv. The radiation dose that patient can be exposed to through the typical length of treatment of 6 months will be about 30 mSv. This extra dose of radiation from one extra scan is limited and acceptable and is not deemed to affect patient' prognosis.

Since the function of most biomarkers (genes and proteins) is not well understood and as there is no normal range for most of them, patients will not be able to use their individual results and we will not be able to interpret anything from a single patient measurement. Hence, we believe that patients will not get any benefit out of being informed of these results, and that it is ethically correct to not inform patients about the results of their biomarkers tests. Patients will be given the option to be contacted with information about the final trial results and whether there is any significance for the individual patient. There will be conducted no new studies of blood samples, which are not described in the project description.

The database and research biobank must be approved by the Danish Data Protection Agency before starting treatment.

The possible advantages the patient will gain from this study outweigh the risks and disadvantages that the patient might experience. Furthermore, the information gained from this study is an important step in developing new treatment regimes in this patient population in great need of new treatment options.

18.3. PATIENT INFORMATION AND INFORMED CONSENT

The subjects should be provided with full and adequate verbal and written information about the objectives, the study outline and possible risks and benefits of participating in the study. The subjects have the right to ask questions about the study and should be given adequate time to make the decision to participate in the study or not. The subject should be clearly informed that the data collected in the study will not identify any subject taking part in the study, following the Law in Personal Data and the EU Data Protection Directive.
The subjects should be informed that it is voluntary to participate and that they can withdraw from the study at any time without giving any particular reason. The subjects should further be informed that a decision not to participate in the study or to withdraw will not be questioned or effect their future medical care or treatment at the clinic.

Written Informed Consent must be obtained from all participating subjects before enrolment in the study. The Informed Consent form should also be signed, at the same occasion, by the investigator who gave the written and verbal information. The Informed Consent form should be filed in the Investigator´s File and one copy should be given to the subject. No trial related procedures can take place unless a written informed consent is obtained. Written Informed Consent regarding inclusion into BIOPAC project must be obtained from all participating subjects before enrolment in the study.

The subjects will consent to: participation in the study; regulatory authorities to gain full access to hospital records, to control the data collected in the study; recording, collecting and processing of data and storing data in a database; and storing of study samples in a biobank.

The Informed Consent form and the written patient information are provided in Appendices 27.2 and 27.3.

19. DATA HANDLING AND RECORD KEEPING

19.1. CASE REPORT FORMS

An electronic eCRF should be completed for each included subject. The subject´s identity must always be kept confidential. The completed original CRF is the sole property of the Investigator and should not be made available to third parties (except for representatives of appropriate authorities).

The Investigator is responsible for ensuring the accuracy, completeness, legibility and timeliness of the data recorded in the CRFs. Database will be situated at the Clinical Trial Unit at the Department of Oncology Herlev & Gentofte University Hospital, Denmark.

19.2. RECORD KEEPING

The Investigator shall keep records of the study to enable evaluations and inspections by regulatory authorities. This includes any original source data related to the study, the subject identification list (with subject numbers, full names and CPR-number), the original signed Informed Consent and CRFs.
The subject identification number will be a XXXXX digit number where the first digit indicates the clinical study site and the three last digits indicate the subjects number at that specific site (e.g. the first subject included at the Department of Oncology, Herlev & Gentofte University Hospital will be 01XXX).

20. INSURANCE

All trial subjects/patients treated with IP are covered by a state insurance scheme (http://patienterstatningen.dk/). The Patient Compensation Association decides compensation claims for patients injured in connection to treatment by the Danish Health Service.

21. PUBLICATION POLICY

Data and results from the study belong to the sponsor and principal investigators. Genis have no influence over the analysis, interpretation or publication of results. After completion of each part of the project (clinical and biomarkers substudies), the results, both positive and negative, and also inconclusive will be presented and published in international journals. All the trial's responsible clinicians should be mentioned in any manuscript or poster, or shown on a slide in connection with lectures. Authors and author order is

determined according to the Vancouver rules. The trial will be made public at the EU Clinical Trials Register.

Patients will only be contacted and informed of the results if they have chosen this.

22. ECONOMY

The study is initiated by the investigators and Genis. Genis will fund (14.000 DKK per patient) this innovative translational project related to a large group of patients with one of the most deadly cancers, and where there are few treatment options. The project will be also supported by the Department of Oncology, Herlev Hospital (partly salary and operating). Genis will cover the cost of T-ChOS™ and placebo.

No funds or sponsors will have any role or influence over the study design, statistical analysis, interpretation of data, manuscript drafts and manuscript revision. None of the researchers has financial interests in the investigation and no financial benefit for the department or its staff will be expected in connection with trial. No remuneration will be paid to patients for participation in the trial.

The drift of the research biobank will be carried out by the Department of Oncology. Registration in the clinical database will be done by the Clinical Trial Unit of the Department of Oncology.
PAXgene tubes will be paid by the Department of Oncology.

23. SUPPLEMENTS

23.1. CHANGES OF THE STUDY PROTOCOL

All changes of the final study protocol must be documented by signed protocol amendments. If substantial changes to the assessments or design of the study are made, the Regulatory Authorities and/or the EC should be notified for review and approval before the changes take place, except for conditions that obviously improves patient's safety.

23.2. APPLICATION TO REGULATORY AUTHORITIES AND ETHICS COMMITTEE

Prior to initiating the clinical study, the investigator will submit an application for authorization to conduct the study, including all required documents, to the Regulatory Authorities in Denmark.
Approval of the study protocol (including any protocol amendment, patient information and Informed Consent forms) must also be obtained from the regional Ethics Committee (EC) before any subject enters into the study. The written approval from the EC should be dated and have an attached list of those persons (with name and positions) present at the EC meeting.
It is the responsibility of the sponsor to report all SARs and SUSARs to the Regulatory Authorities, Danish Data Protection Agency and the IEC. Further the sponsor will report when the study is completed or if the study for any reason stops prematurely.

23.3. STAFF INFORMATION

It is the responsibility of the Investigator to ensure that all personnel involved in the study are fully informed of all relevant aspects of the study, including detailed knowledge of and training in all procedures to be followed.
The signature and delegation log should be continuously updated and signed by the Investigator.

23.4. CRITERIA FOR TERMINATION OF THE STUDY

The chief investigator has the right to discontinue the study prior to inclusion of the intended numbers of subjects.
The study will be prematurely discontinued in the following cases:

- Unexpectedly high proportion of AEs that are possibly or probably related to the study treatment.
- New findings about the investigational product(s) that changes the benefit/risk ratio.
- Unacceptable low Investigator, sponsor or subject compliance.
- Recruitment of eligible subjects is far too low.

23.5. STUDY TIME SCHEDULE

Anticipated start of study: June 1, 2016
Estimated recruitment period: June 1, 2016 – May 31, 2019
End of follow-up: May 31, 2021

24. REFERENCES

1. Ferlay J, Soerjomataram I, Dikshit R, et al.: Cancer incidence and mortality worldwide: sources, methods and major patterns in GLOBOCAN 2012. Int J Cancer 136:E359-E386, 2015

2. Ferlay J, Steliarova-Foucher E, Lortet-Tieulent J, et al.: Reprint of: Cancer incidence and mortality patterns in Europe: Estimates for 40 countries in 2012. Eur J Cancer 51:1201-1202, 2015

3. Siegel RL, Miller KD, Jemal A: Cancer statistics, 2016. CA Cancer J Clin 66:7-30, 2016

4. Hidalgo M: Pancreatic cancer. N Engl J Med 362:1605-1617, 2010

5. Ryan DP, Hong TS, Bardeesy N: Pancreatic adenocarcinoma. N Engl J Med 371:2140-2141, 2014

6. Oettle H, Neuhaus P, Hochhaus A, et al.: Adjuvant chemotherapy with gemcitabine and long-term outcomes among patients with resected pancreatic cancer: the CONKO-001 randomized trial. JAMA 310:1473-1481, 2013

7. Neoptolemos JP, Dunn JA, Stocken DD, et al.: Adjuvant chemoradiotherapy and chemotherapy in resectable pancreatic cancer: a randomised controlled trial. Lancet 358:1576-1585, 2001

8. Neoptolemos JP, Stocken DD, Bassi C, et al.: Adjuvant chemotherapy with fluorouracil plus folinic acid vs gemcitabine following pancreatic cancer resection: a randomized controlled trial. JAMA 304:1073-1081, 2010

9. Neoptolemos JP, Palmer D, Ghaneh P, et al.: ESPAC-4: A multicenter, international, open-label randomized controlled phase III trial of adjuvant combination chemotherapy of gemcitabine (GEM) and capecitabine (CAP) versus monotherapy gemcitabine in patients with resected pancreatic ductal adenocarcinoma. J Clin Oncol 34, abstr LBA4006, 2016 (abstr)

10. Johansen JS, Schultz NA, Jensen BV: Plasma YKL-40: a potential new cancer biomarker? Future Oncol 5:1065-1082, 2009

11. Faibish M, Francescone R, Bentley B, et al.: A YKL-40-neutralizing antibody blocks tumor angiogenesis and progression: a potential therapeutic agent in cancers. Mol Cancer Ther 10:742-751, 2011

12. Lee CG, Da Silva CA, Dela Cruz CS, et al.: Role of chitin and chitinase/chitinase-like proteins in inflammation, tissue remodeling, and injury. Annu Rev Physiol 73:479-501, 2011

13. Shao R: YKL-40 acts as an angiogenic factor to promote tumor angiogenesis. Front Physiol 4:122, 2013

14. Shao R, Francescone R, Ngernyuang N, et al.: Anti-YKL-40 antibody and ionizing irradiation synergistically inhibit tumor vascularization and malignancy in glioblastoma. Carcinogenesis 35:373-382, 2014

15. Johansen JS, Hoyer PE, Larsen LA, et al.: YKL-40 protein expression in the early developing human musculoskeletal system. J Histochem Cytochem 55:1213-1228, 2007

16. Chen CC, Llado V, Eurich K, et al.: Carbohydrate-binding motif in chitinase 3-like 1 (CHI3L1/YKL-40) specifically activates Akt signaling pathway in colonic epithelial cells. Clin Immunol 140:268-275, 2011

17. Lee CG, Hartl D, Lee GR, et al.: Role of breast regression protein 39 (BRP-39)/chitinase 3-like-1 in Th2 and IL-13-induced tissue responses and apoptosis. J Exp Med 206:1149-1166, 2009

18. Libreros S, Iragavarapu-Charyulu V: YKL-40/CHI3L1 drives inflammation on the road of tumor progression. J Leukoc Biol, 2015

19. He CH, Lee CG, Dela Cruz CS, et al.: Chitinase 3-like 1 regulates cellular and tissue responses via IL-13 receptor alpha2. Cell Rep 4:830-841, 2013

20. Libreros S, Garcia-Areas R, Shibata Y, et al.: Induction of proinflammatory mediators by CHI3L1 is reduced by chitin treatment: decreased tumor metastasis in a breast cancer model. Int J Cancer 131:377-386, 2012

21. Suzuki K, Mikami T, Okawa Y, et al.: Antitumor effect of hexa-N-acetylchitohexaose and chitohexaose. Carbohydr Res 151:403-408, 1986

22. Tokoro A, Tatewaki N, Suzuki K, et al.: Growth-inhibitory effect of hexa-N-acetylchitohexaose and chitohexaose against Meth-A solid tumor. Chem Pharm Bull (Tokyo) 36:784-790, 1988

23. Tsukada K, Matsumoto T, Aizawa K, et al.: Antimetastatic and growth-inhibitory effects of N-acetylchitohexaose in mice bearing Lewis lung carcinoma. Jpn J Cancer Res 81:259-265, 1990

24. Shen KT, Chen MH, Chan HY, et al.: Inhibitory effects of chitooligosaccharides on tumor growth and metastasis. Food Chem Toxicol 47:1864-1871, 2009

25. Maeda Y, Kimura Y: Antitumor effects of various low-molecular-weight chitosans are due to increased natural killer activity of intestinal intraepithelial lymphocytes in sarcoma 180-bearing mice. J Nutr 134:945-950, 2004

26. Harish Prashanth KV, Tharanathan RN: Depolymerized products of chitosan as potent inhibitors of tumor-induced angiogenesis. Biochim Biophys Acta 1722:22-29, 2005

27. Hezel AF, Kimmelman AC, Stanger BZ, et al.: Genetics and biology of pancreatic ductal adenocarcinoma. Genes Dev 20:1218-1249, 2006

28. Waddell N, Pajic M, Patch AM, et al.: Whole genomes redefine the mutational landscape of pancreatic cancer. Nature 518:495-501, 2015

29. Ballehaninna UK, Chamberlain RS: The clinical utility of serum CA 19-9 in the diagnosis, prognosis and management of pancreatic adenocarcinoma: An evidence based appraisal. J Gastrointest Oncol 3:105-119, 2012

30. Tempero MA, Uchida E, Takasaki H, et al.: Relationship of carbohydrate antigen 19-9 and Lewis antigens in pancreatic cancer. Cancer Res 47:5501-5503, 1987

31. Mann DV, Edwards R, Ho S, et al.: Elevated tumour marker CA19-9: clinical interpretation and influence of obstructive jaundice. Eur J Surg Oncol 26:474-479, 2000

32. Marrelli D, Caruso S, Pedrazzani C, et al.: CA19-9 serum levels in obstructive jaundice: clinical value in benign and malignant conditions. Am J Surg 198:333-339, 2009

33. Schultz NA, Christensen IJ, Werner J, et al.: Diagnostic and Prognostic Impact of Circulating YKL-40, IL-6, and CA 19.9 in Patients with Pancreatic Cancer. PLoS One 8:e67059, 2013

34. Bettegowda C, Sausen M, Leary RJ, et al.: Detection of circulating tumor DNA in early- and late-stage human malignancies. Sci Transl Med 6:224ra24, 2014

35. Heitzer E, Ulz P, Geigl JB: Circulating tumor DNA as a liquid biopsy for cancer. Clin Chem 61:112-123, 2015

36. Almoguera C, Shibata D, Forrester K, et al.: Most human carcinomas of the exocrine pancreas contain mutant c-K-ras genes. Cell 53:549-554, 1988

37. Hruban RH, van Mansfeld AD, Offerhaus GJ, et al.: K-ras oncogene activation in adenocarcinoma of the human pancreas. A study of 82 carcinomas using a combination of mutant-enriched polymerase chain reaction analysis and allele-specific oligonucleotide hybridization. Am J Pathol 143:545-554, 1993

38. Guo Y, Xu F, Lu T, et al.: Interleukin-6 signaling pathway in targeted therapy for cancer. Cancer Treat Rev 38:904-910, 2012

39. Ataie-Kachoie P, Pourgholami MH, Richardson DR, et al.: Gene of the month: Interleukin 6 (IL-6). J Clin Pathol 67:932-937, 2014

40. Ancrile B, Lim KH, Counter CM: Oncogenic Ras-induced secretion of IL6 is required for tumorigenesis. Genes Dev 21:1714-1719, 2007

41. Middleton K, Jones J, Lwin Z, et al.: Interleukin-6: an angiogenic target in solid tumours. Crit Rev Oncol Hematol 89:129-139, 2014

42. Rowley M, Van NB: Activation of N-ras and K-ras induced by interleukin-6 in a myeloma cell line: implications for disease progression and therapeutic response. Oncogene 21:8769-8775, 2002

43. Scheller J, Ohnesorge N, Rose-John S: Interleukin-6 trans-signalling in chronic inflammation and cancer. Scand J Immunol 63:321-329, 2006

44. Demaria S, Pikarsky E, Karin M, et al.: Cancer and inflammation: promise for biologic therapy. J Immunother 33:335-351, 2010

45. Pietras K, Ostman A: Hallmarks of cancer: interactions with the tumor stroma. Exp Cell Res 316:1324-1331, 2010

46. Hanahan D, Weinberg RA: Hallmarks of cancer: the next generation. Cell 144:646-674, 2011

47. Hanahan D, Coussens LM: Accessories to the crime: functions of cells recruited to the tumor microenvironment. Cancer Cell 21:309-322, 2012

48. Grivennikov SI, Greten FR, Karin M: Immunity, inflammation, and cancer. Cell 140:883-899, 2010

49. Rakoff-Nahoum S: Why cancer and inflammation? Yale J Biol Med 79:123-130, 2006

50. Holmer R, Goumas FA, Waetzig GH, et al.: Interleukin-6: a villain in the drama of pancreatic cancer development and progression. Hepatobiliary Pancreat Dis Int 13:371-380, 2014

51. Strassmann G, Fong M, Kenney JS, et al.: Evidence for the involvement of interleukin 6 in experimental cancer cachexia. J Clin Invest 89:1681-1684, 1992

52. Musselman DL, Miller AH, Porter MR, et al.: Higher than normal plasma interleukin-6 concentrations in cancer patients with depression: preliminary findings. Am J Psychiatry 158:1252-1257, 2001

53. Jacober ML, Mamoni RL, Lima CS, et al.: Anaemia in patients with cancer: role of inflammatory activity on iron metabolism and severity of anaemia. Med Oncol 24:323-329, 2007

54. Ebrahimi B, Tucker SL, Li D, et al.: Cytokines in pancreatic carcinoma: correlation with phenotypic characteristics and prognosis. Cancer 101:2727-2736, 2004

55. Nixon AB, Pang H, Starr MD, et al.: Prognostic and predictive blood-based biomarkers in patients with advanced pancreatic cancer: results from CALGB80303 (Alliance). Clin Cancer Res 19:6957-6966, 2013

56. Bellone G, Smirne C, Mauri FA, et al.: Cytokine expression profile in human pancreatic carcinoma cells and in surgical specimens: implications for survival. Cancer Immunol Immunother 55:684-698, 2006

57. Mitsunaga S, Ikeda M, Shimizu S, et al.: Serum levels of IL-6 and IL-1beta can predict the efficacy of gemcitabine in patients with advanced pancreatic cancer. Br J Cancer 108:2063-2069, 2013

58. Mueller TC, Burmeister MA, Bachmann J, et al.: Cachexia and pancreatic cancer: are there treatment options? World J Gastroenterol 20:9361-9373, 2014

59. Je JY, Kim SK: Chitooligosaccharides as potential nutraceuticals: production and bioactivities. Adv Food Nutr Res 65:321-336, 2012

60. Gesto DS, Cerqueira NM, Fernandes PA, et al.: Gemcitabine: a critical nucleoside for cancer therapy. Curr Med Chem 19:1076-1087, 2012

61. Mini E, Nobili S, Caciagli B, et al.: Cellular pharmacology of gemcitabine. Ann Oncol 17 Suppl 5:v7-12, 2006

25. COORDINATING PRINCIPAL INVESTIGATOR'S AGREEMENT

EudraCT number:	2014-001163-12
Title of the study:	A Single Center, Randomized, Double-Blind, Placebo-Controlled Phase 2 Study of Gemcitabine (GEM) and Capecitabine (CAP) with or without T-ChOSTM as adjuvant therapy in patients with surgically resected pancreatic cancer.

I, the undersigned, have read and understand the protocol specified above and agree that it contains all necessary information for conducting the study. The study protocol, the Clinical Study Agreement and the additional information given will serve as a basis for co-operation in this study.

I agree to conduct the study according to this protocol and according to the ethical principles that have their origin in the Declaration of Helsinki and that are consistent with Good Clinical Practice and the applicable laws and regulations.

**Sponsor**<br>DORTE LISBET NIELSEN, MD, DMSc	______________________<br>Signature<br><br>______________________<br>Date

26. PRINCIPAL INVESTIGATOR'S AGREEMENT

EudraCT number:	2014-001163-12
Title of the study:	A Single Center, Randomized, Double-Blind, Placebo-Controlled Phase 2 Study of Gemcitabine (GEM) and Capecitabine (CAP) with or without T-ChOSTM as adjuvant therapy in patients with surgically resected pancreatic cancer.

I, the undersigned, have read and understand the protocol specified above and agree that it contains all necessary information for conducting the study. The study protocol, the Clinical Study Agreement and the additional information given will serve as a basis for co-operation in this study.

I agree to conduct the study according to this protocol and according to the ethical principles that have their origin in the Declaration of Helsinki and that are consistent with Good Clinical Practice and the applicable laws and regulations.

**Principal Investigator**<br><br>Name:________________________________<br><br>Title:________________________________<br><br>Work Address:________________________<br><br>____________________________________<br><br>____________________________________	____________________________________<br>Signature<br><br>____________________________________<br>Date

27. APPENDICES

27.1 PROTOCOL SUMMARY (IN DANISH), VERSION 1.0, 2015-10-21

27.2 PATIENT INFORMATION SHEET (IN DANISH), VERSION 1.0, 2015-10-21

27.3 INFORMED CONSENT (IN DANISH), VERSION 1.0, 2015-10-21

27.4 FIRST CONTACT AND INFORMED CONSENT GUIDANCE (IN DANISH), VERSION 1.0, 2015-10-21

27.5 NOTIFICATION OF SERIOUS ADVERSE EVENT (SAE), VERSION, 21.01.2014

27.6 EORTC QUALITY OF LIFE QUESTIONNAIRE C30 (QLQ-C30) VERSION 3.0

27.1. APPENDIX: PROTOCOL SUMMARY (IN DANISH), VERSION 1.0, 2015-10-21

Protokolresume – Pancreas Cancer T-ChOS adjuverende

Et enkelt center, randomiseret, dobbelt blindt, placebo-kontrolleret fase II undersøgelse af gemcitabin og capecitabin med eller uden T-ChOS™ til patienter efter operation for kræft i bugspytkirtlen

Forsøgsansvarlig: Afdelingslæge Inna Chen.
Sponsor: Professor, overlæge, dr. med., ph.d. Dorte Nielsen.
Onkologisk afdeling, Herlev Hospital, Herlev Ringvej 75, 2730 Herlev.

Forsøget vedrører behandling med gemcitabin og T-ChOS[TM] eller placebo til patienter efter operation for kræft i bugspytkirtlen (pancreas cancer).

Baggrund
Der bliver hvert år i Danmark diagnosticeret ca. 950 patienter med kræft i bugspytkirtlen (pancreas cancer). Kun ca. 20 % af patienterne er operable. I dag anbefaler man 6 måneders forebyggende (adjuverende) kemobehandling med gemcitabin og capecitabin til patienter efter operation for kræft i bugspytkirtlen. Desværre er virkningen af gemcitabin og capecitabin beskeden, idet mange patienter hurtigt får tilbagefald af kræftsygdommen og kun ca. 30 % af patienterne er i live 5 år efter operationen. Vi ønsker derfor i dette forsøg at undersøge om et kosttilskud Benecta (T-ChOS[TM]) er effektivt i kombination med gemcitabin og capecitabin. T-ChOS[TM] har i prækliniske studier vist at kunne blokere proteinet YKL-40, der stimulerer vækst af forskellige typer kræft.

Formål med forsøget
- At undersøge hvordan T-ChOS[TM] behandling i kombination med gemcitabin og capecitabin virker på sygdommen
- At beskrive de bivirkninger der opstår ved T-ChOS[TM] behandling

Vi håber, at vores resultater i fremtiden vil kunne anvendes til at behandle patienter efter operation for kræft i bugspytkirtlen og derved vil kunne komme patienter med kræft i bugspytkirtlen til gavn fremover.

Behandling og undersøgelser
Før Behandling: Før behandling vil der blive foretaget CT-skanning. I behandlingsforløbet vil sygdommen blive fulgt med CT-skanning efter 3 og 6 måneders gemcitabin og capecitabin behandling. Derefter vil patienterne følges med CT-skanning og klinisk kontrol hver 3. måned det første år, hver 4. måned år 2. og 3, hver 6 måned år 4. og 5.

Rutine og projektblodprøver: Patienterne vil før og under behandlingen få taget rutine blodprøver (10 ml) (hæmatologi, lever- og nyretal, elektrolytter, glukose, CRP, og CA 19-9) før hver serie og i forbindelse med CT-skanning. Desuden vil patienterne få taget projektblodprøver (59 ml) før første og anden serie, samt i forbindelse med CT-skanning iht. BIOPAC projektet. De indsamles blod- og vævsprøver vil opbevares i BIOPAC forskningsbiobanken på Onkologisk Afdeling Herlev og Gentofte Hospital i op til 20 år efter afslutningen af undersøgelsen. Evt. overskydende restmateriale (blod- og vævsprøver) vil efter 20 år overgå i en biobank. Såfremt der senere skal laves nye undersøgelser på dette restmateriale vil der søges om tilladelse til disse nye analyser hos den Videnskabs Etiske Komite i form af tillægsprotokoller. Der vil anvendes

samme standard operationelle procedurer (SOP) for indsamling af blod- og vævsprøver, håndtering og opbevaring af disse prøver, som beskrevet i BIOPAC projektet ("BIOmarkers in patients with Pancreatic Cancer"; VEK ref. KA-20060113; j.nr. 2006-41-6848 og HGH-2015-027, I-Suite nr: 03960). Prøverne og en liste med identifikation vil blive opbevaret sikkert og hver for sig i henhold til regler fra Datatilsynet. Fra patientens journal vil der indhentes kliniske oplysninger, blodprøvesvar, patologisvar og skanningssvar samt oplysninger vedr. den medicinske behandling og evt. bivirkninger. Disse oplysninger vil blive behandlet strengt fortroligt i en database tilknyttet forskningsprojektet i henhold til regler fra Datatilsynet.

Kliniske oplysninger: Fra patientens journal vil der indhentes kliniske oplysninger, blodprøvesvar, patologisvar og CT-skanningssvar samt oplysninger vedr. behandlingen og evt. bivirkninger. Disse oplysninger vil blive behandlet strengt fortroligt i en database tilknyttet forskningsprojektet.

Forsøgsbehandlingen

Gemcitabin (Gemzar®)
Gemcitabin er et velkendt og godkendt kemoterapi til behandling af mange kræftformer, bl.a. kræft i bugspytkirtlen. Gemcitabin gives i en blodåre dag 1, dag 8 og dag 15, hver 4. uge (kaldet en serie).

Capecitabin
Capecitabin er et velkendt og godkendt kemoterapi til behandling af mange kræftformer, bl.a. kræft i bugspytkirtlen i kombinatuon med gemcitabin. Capecitabin-tabletter tages morgen og aften i 21 dage, begyndende om aftenen efter kemoterapien. Herefter holdes en uges pause med tabletterne., hver 4. uge (kaldet en serie).

T-ChOS™
I dette forsøg ønsker vi at undersøge om kapsel behandling med T-ChOS™ givet i kombination med gemcitabin og capecitabin kan forbedre behandlingen af patienter med kræft i bugspytkirtlen. T-ChOS™ er udviklet af rejeskaller og gives som kapsel behandling hver morgen (2 kapsler af 300 mg).

Der vil være to behandlingsregimer i forsøget:
- Behandlingsregime 1: Gemcitabin og capecitabin i kombination med T-ChOS™
- Behandlingsregime 2: Gemcitabin og capecitabin i kombination med et ikke aktivt stof (placebo)

Hvilken behandling patienten skal have vil blive afgjort ved lodtrækning, og denne lodtrækning vil blive foretaget af en computer. Hvilket betyder, at det være helt tilfældigt, hvilken behandling patienten får tildelt. Ingen af de forsøgsansvarlige vil vide om patienten får det aktive T-ChOS™ eller det ikke aktive teststof (placebo). Det er apoteket, der blinder kapsler.

Bivirkninger til kemoterapi (gemcitabin og capecitabin)
Træthed.
Kvalme og opkastning (for at forebygge disse gener gives kvalmestillende kapsler).
Påvirkning af knoglemarven kan vise sig i form af blodmangel, infektion og feber. Slimhinder i mund, skede, mave og tarm kan blive irriterede og dermed mere følsomme. Hermed diarré og stomatitis,

Specielt ved Gemcitabin: Influenzalignende symptomer med feber, kulderystelser og muskelømhed kan forekomme i lettere grad efter behandling med Gemcitabin. Det varer sjældent over 2 døgn, og kan lindres med Panodil.

Specielt ved Capecitabin: Påvirkninger af hjertet. Dette kan opleves som brystsmerter, rytmeforstyrrelser og åndenød. Hand-and-Foot Syndrome. Huden kan blive tør, rød, revne og skalle af.

Bivirkninger til T-ChOS™ behandlingen

Nedenstående hændelser, der er set i tidligere forsøg med T-ChOS™, er ikke vurderet at have relation til behandlingen med T-ChOS:

Milde	Appetitløshed, kvalme, øget sexual lyst, åndenød, halsbrand, tidlig mæthed.
Moderate	Prikkende og stikkende fornemmelse i knæ regionen, øget appetit, mundtørhed, øget tørst.
Alvorlige	Øgede knæsmerter, udslet på mave og brystregion, hudkløe, svimmelhed, rystelser i kroppen.

Det kan ikke udelukkes, at der kan forekomme andre bivirkninger. Hvis vi opdager bivirkninger, som vi ikke allerede har beskrevet, vil patienten blive orienteret med det samme.

Kombinationen af gemcitabin og capecitabin med T-ChOS™

Kombinationen af gemcitabin og capecitabin med T-ChOS™ er ukendt. Gemcitabin og capecitabin behandling giver få bivirkninger, så tillæg af T-ChOS™ forventes ikke at ville give yderligere moderate eller alvorlige bivirkninger. Dosis af gemcitabin og capecitabin er lagt således at risikoen for knoglemarvssuppresion er minimal.

Varighed af behandlingen

Gemcitabin og capecitabin behandlingen gives i 6 måneder, men stopper før hvis patienten får tilbagefald af sygdommen. T-ChOS™/placebo behandlingen gives dagligt i de 6 måneder, som patienten får gemcitabin og capecitabin behandling. T-ChOS™/placebo behandlingen fortsætter derefter indtil der er tegn på tilbagefald af sygdommen i højst 5 år. Behandlingen med gemcitabin, capecitabin og T-ChOS™/placebo vil dog stoppe før såfremt patienten får for mange bivirkninger eller patienten selv ønsker at stoppe behandlingen.

Inklusion

Patienter med kræft i bugspytkirtlen som skal starte forebyggende behandling med gemcitabin og capecitabin efter operation for kræft i bugspytkirtlen.

Etiske overvejelser

I denne undersøgelse behandles patienter, der er opereret for kræft i bugspytkirtlen. Den behandling de vil modtage er dels kendte stoffer (gemcitabin og capecitabin) med begrænsede bivirkninger. De mulige bivirkninger til T-ChOS™ er efter vores mening milde til moderate og tolerable.

De ansvarlige investigatorer vil sørge for, at studiet bliver udført i overensstemmelserne med Helsinki-deklarationen og landets love og vedtægter. Protokollen vil blive godkendt af den lokale Videnskabsetiske Komité, Lægemiddelstyrelsen og Datatilsynet.

Patienterne oplyses ikke om undersøgelsesresultaterne, da disse ikke vil få nogen behandlingsmæssige konsekvenser for patienterne. Baggrunden herfor er, at betydningen af eventuelle fund ikke er afklaret. Patienterne vil dog have mulighed for at blive kontaktet med information om forsøgets endelige resultater og om en eventuel betydning for den enkelte patient.

Patienter og projektperiode

Det forventes, at den første patient kan inkluderes i juni 2016 og at alle 180 patienter er inkluderet i juni 2019. Hver patient følges til død eller i minimum 2 år, dvs. studiet slutter i juni 2021.

Sponsor og økonomi

Undersøgelsen er initieret af investigatorerne.

Undersøgelsen er støttet med ~14.000 kroner per patient fra Genis. Der vil desuden søges om økonomisk støtte fra offentlige såvel private danske fonde til gennemførelsen af projektet. Ingen fonde eller sponsorer vil få nogen rolle i eller indflydelse på studiets design, statistiske analyser, fortolkning af data, manuskriptudkast eller manuskriptrevision.

Ingen af forskerne har økonomiske interesser i undersøgelsen, og der er i forbindelse med dette forsøg ingen økonomisk gevinst for afdelingen eller dets personale. Der udbetales intet vederlag til patienterne for deltagelse i forsøget.

Patienten vil være dækket af hospitalets forsikring, hvis der mod forventning skulle opstå skade forårsaget af forsøget.

Offentliggørelse

Efter afslutning af undersøgelsen udfærdiges publikation vedr. studiet, som vil blive offentliggjort i internationale tidsskifter. Positive, negative og inkonklusive resultater vil blive offentliggjort. Patienterne vil kun blive kontaktet og informeret om resultaterne såfremt de har valgt dette. Forfattere og forfatterrækkefølge bestemmes i henhold til Vancouver-reglerne.

Deltagerinformation om deltagelse i et sundhedsvidenskabeligt forsøg

En randomiseret, dobbelt blindt placebo-kontrolleret fase II undersøgelse af T-ChOS™ eller placebo i kombination med standardbehandling med Gemcitabin og Capecitabin, som adjuverende (forebyggende) behandling til patienter, der er opereret for kræft i bugspytkirtlen

Onkologisk Afdeling
Herlev Hospital

Forsøgsansvarlig /sponsor	Dorte Nielsen
Hospital	Herlev
Adresse	Herlev Ringvej 75, 2730 Herlev
Telefonnummer	[redacted]
E-mail adresse	[redacted]
Forsøgspersonens nummer	01 _ _ _

En randomiseret, dobbelt blindt placebo-kontrolleret fase II undersøgelse af T-ChOS™ eller placebo i kombination med standardbehandling med Gemcitabin og Capecitabin, som adjuverende (forebyggende) behandling til patienter, der er opereret for kræft i bugspytkirtlen

Vi vil gerne spørge dig, om du vil deltage i et sundhedsvidenskabeligt forsøg, der skal undersøge virkningen af et kosttilskud med navnet T-ChOS™ eller placebo (et ikke aktivt stof) i kombination med de kendte stoffer Gemcitabin og Capecitabin til patienter, der er opereret for kræft i bugspytkirtlen (forebyggende behandling).

Forsøget starter i juni 2016 og forventes at slutte i juni 2019. Det er planlagt, at der skal deltage 180 patienter i forsøget. Forsøget er iværksat af læger i Onkologisk Afdeling på Herlev Hospital. Der deltager ikke andre hospitaler end Herlev Hospital.

Forsøgsansvarlig, repræsentanter fra GCP- enhederne, Lægemiddelstyrelsen, Videnskabsetisk Komité, Fødevarestyrelsen og Genis kan få videregivet oplysninger fra den behandlingsansvarlige læge.

Denne deltagerinformation forklarer, hvad forsøget indebærer og din rolle som mulig deltager. Den skriftlige information er et supplement til den mundtlige information. Du er velkommen til at tage en pårørende med til samtalen. Læs venligst denne information grundigt, og tøv endelig ikke med at stille spørgsmål. Hvis du beslutter dig for at deltage i dette forsøg, skal du underskrive samtykkeerklæringen og fuldmagten. Begge dele finder du bagerst i denne information.

Du hat ret til betænkningstid, før du beslutter, om du vil deltage. Vi opfordrer dig til at læse ”Forsøgspersoners rettigheder i et sundhedsvidenskabeligt forskningsprojekt”, som du ligeledes finder bagerst i denne information.

Frivillig deltagelse

Din deltagelse i dette forskningsforsøg er frivillig. Du behøver ikke at deltage, og selv om du beslutter at deltage, kan du ændre mening på ethvert tidspunkt og trække dit tilsagn om deltagelse tilbage, uden at det får indflydelse på dit forhold til Onkologisk Afdeling, eller behandlingen af din sygdom. Den forsøgsansvarlige læge eller Sundhedsstyrelsen kan også være nødsaget til at trække dig ud af forsøget eller stoppe forsøget på grund af en eller flere af følgende årsager:

- Du har brug for behandling, som ikke tillades i forsøget.
- Den forsøgsansvarlige læge vurderer, at din deltagelse kan skade dig.
- Manglende overholdelse af forsøgslægens og/eller forsøgspersonalets anvisninger.
- Der opstår alvorlige bivirkninger.
- Forsøget aflyses.

- Hvis du bliver gravid.
- Uventede omstændigheder.

Hvis det bliver nødvendigt at trække dig ud af forsøget, vil forsøgslægen diskutere dette med dig.

Baggrund og formål med forsøget

Standardbehandlingen til patienter, der er opereret for kræft i bugspytkirtlen, hvor man har fjernet alt synligt kræftvæv, er kemoterapi, med velkendte stoffer Gemcitabin og Capecitabin. I dette forsøg ønsker vi at finde ud af, om T-ChOS™ givet i kombination med Gemcitabin og Capecitabin kan forbedre muligheden for, at din kræftsygdom ikke vender tilbage. T-ChOS™ er ikke et lægemiddel, men et kosttilskud, der er udviklet af rejeskaller. Cellestudier foretaget i laboratoriet har vist, at T ChOS™ kan reducere væksten af kræftceller, men vi ved endnu ikke om denne virkning kan overføres til mennesker. Vi forventer, at projektet kan bringe ny og værdifuld viden om dette. Der er 1/2 chance for, at du får en af følgende behandlinger:

- 1/2 af patienterne vil få Gemcitabin og Capecitabin i kombination med T-ChOS™
- 1/2 af patienterne vil få Gemcitabin og Capecitabin i kombination med et ikke aktivt stof (placebo)

Det vil blive afgjort ved lodtrækning, hvilken behandling du skal have. Lodtrækningen vil blive foretaget af en computer. Det betyder, at det vil være helt tilfældigt, hvilken behandling du får tildelt. Ingen af de forsøgsansvarlige ved, om du får det aktive T-ChOS™, eller om du får et ikke aktivt teststof (placebo). Det er apoteket, der blinder kapslerne.
Desuden har forsøget også til formål at registre og beskrive bivirkningerne af behandlingen.

Behandlingen

Behandlingens varighed

Du får behandling med Gemcitabin på dag 1, dag 8 og dag 15 hver 4. uge. Capecitabin-tabletterne skal du tage morgen og aften i 21 dage, efterfulgt af en uges pause, hver 4. uge (kaldet en serie). T-ChOS™ / placebo vil gives hver dag. Se skemaet, der viser behandlingen nederst på siden. Behandlingens varighed er på 6 måneder. Behandlingen med T-ChOS™ **/** placebo vil fortsætte så længe skanningerne viser, at du er sygdomsfri i højst 5 år, eller så længe du selv ønsker det og kan tåle behandlingen. Mens du er sygdomsfri, vil du blive fulgt med CT-skanning efter 3 og 6 måneders gemcitabin og capecitabin behandling. Derefter vil du følges med CT-skanning hver 3. måned det første år, hver 4. måned år 2. og 3, hver 6 måned år 4. og 5.

Behandlingen får du ambulant. Det vil sige, at du møder i ambulatoriet, hvor du får Gemcitabin i en blodåre i armen. Behandlingen tager ca. 30 – 45 min. Capecitabin-tabletter tages morgen og aften. T-ChOS™ / placebo gives som kapsler hver dag. Kapslerne tages om morgenen med et glas vand ½ time før morgenmåltidet. Behandlingen vil foregå uden pauser.
Du skal føre dagbog over, hvilke tider du tager T-ChOS™ på. Du får udleveret dagbogen på afdelingen.

	Dag 0	Dag 1	Dag 8	Dag 15
Læge-samtale	X		X	
Blodprøver<br>EKG (kun dag 0)	X		X	
Udfylde livskvalitetsskema	X			
Behandling med Gemcitabin		X	X	X
Behandling med Capecitabin		Tabletter tages morgen og aften i 21 dage		
Behandling med T-ChOS™ eller placebo		Kapslerne tages hver dag fra dag 1		
Dette skema viser én serie. En serie svarer til en periode på 4 uger, hvor der gives Gemcitabin på dag 1, dag 8 og dag 15. Herefter skal der ikke gives behandling med Gemcitabin i 14 dage.				

Undersøgelser

Før behandlingen

Før du kan indgå i forsøget, skal du have lavet forskellige undersøgelser, der bl.a. skal sikre, at du kan tåle behandlingen. Du bliver undersøgt af en læge og får taget blodprøver og et hjertekardiogram. Du får desuden foretaget en CT-skanning af dit bryst og din mave.
Hvis du er kvinde i den fødedygtige alder, skal du også have foretaget en graviditetstest.
Derudover skal du udfylde et livskvalitetsskema, før du starter behandlingen i forsøget og hver 12. uge mens du får kemoterapi. Derefter hver 3. måned det første år, hver 4. måned år 2. og 3, hver 6 måned år 4. og 5.

Blodprøver

Vi ønsker hele tiden at øge vores viden om kræft i bugspytkirtlen. Det gælder både viden om sygdommen og viden om den bedste behandling. Vi kender endnu ikke årsagen til, at bugspytkirtelkræft opstår. Der er dog enighed om, at bugspytkirtelkræft opstår og udvikler sig på grund af ændringer i kirtlens arvelige egenskaber.
Vi ønsker at give vore patienter den bedst mulige behandling, derfor beder vi dig om tilladelse til at tage ekstra blodprøver i forbindelse med dette forsøg. De ekstra prøver bliver taget, når du alligevel skal have taget blodprøver. Det vil sige før behandlingsstart, før 2. behandlingsserie, og i forbindelse med hver CT-scanning. Ved hver ekstra blodprøve bliver der udtaget 59 ml blod, hvilket ikke får indflydelse på dit helbred.
Blodprøverne bliver frosset ned med henblik på senere analyse. Blodprøverne vil blive undersøgt for ændringer i arvemateriale (gener), æggehvidestoffer (proteiner) og metaboliter, også kaldet biomarkører.
Undersøgelserne af blodprøverne kan være med til at kaste nyt lys over, hvordan bugspytkirtelkræft udvikler sig.
Ovennævnte analyser vil ikke få direkte betydning for behandlingen af din sygdom, men de vil kunne give nyttig information for fremtidig behandling.
Blodprøverne bliver opbevaret i BIOPAC forskningsbiobanken i højst 20 år. Eventuelt overskydende restmateriale vil efter 20 år blive opbevaret i en biobank.
Du kan ikke deltage i forsøget, hvis du ikke ønsker, at der tages ekstra blodprøver.

Risiko ved forsøgsprocedurer

CT-skanning: Alle mennesker udsættes for en lille mængde uundgåelig stråling hvert år. Noget af denne stråling kommer fra rummet, og noget kommer fra naturligt forekommende radioaktive former for vand og mineraler. I dette forsøg vil du blive udsat for en vis dosis røntgenstråler i forbindelse med CT-skanninger. Stråledosis for hver CT-skanning er ca. 10 mSv. Den stråledosis, som du kan blive udsat for gennem den typiske behandlingslængde på 6 måneder, vil udgøre ca. 30 mSv. Denne dosis er begrænset og acceptabel, og vurderes ikke at have betydning for din prognose. Den strålingsdosis, som vi har omtalt, omfatter kun den stråling, som modtages fra dette forsøg. Den omfatter ikke stråling, som du muligvis får fra andre undersøgelser i forbindelse med din sygdom.

For bedre at kunne se de indre strukturer i kroppen under CT-skanningen får du indsprøjtet noget kontrastvæske ("farvestof"). I sjældne tilfælde kan nålen (kateteret) glide ud af blodåren, så det indsprøjtede kontraststof kommer ud i vævet og giver lokal smerte. Det behandles som regel med kompres. Der kan forekomme reaktioner, som f.eks. metallisk smag i munden, kvalme, opkastning og nældefeber. Dette er som regel begrænset. I meget sjældne tilfælde kan der opstå vejrtrækningsproblemer, blodtryksfald og svimmelhed, som kræver behandling. Alvorlige reaktioner, som medfører dødsfald, er ekstremt sjældne. Deltagere, der i forvejen har allergi, har en større risiko for at få reaktioner end deltagere uden allergi. Hvis det er relevant, kan vi give forebyggende medicin efter samråd med lægen for at nedsætte risikoen for allergiske reaktioner.

Blodprøver: Hos de fleste giver nålestik ikke problemer. Der kan dog forekomme let blødning og et blåt mærke ved indstiksstedet.

Bivirkninger

Behandlingen kan give bivirkninger. Vi forventer ikke at se alle bivirkninger hos dig, men kan dog ikke på forhånd udelukke andre bivirkninger end de, der er nævnt her. For at vi kan forebygge og behandle eventuelle bivirkninger, er det vigtigt, at du oplyser os om de gener, du har i forbindelse med behandlingen.

Kemoterapi (Gemcitabin og Capecitabin)

Træthed: Du kan opleve betydelig træthed og et behov for at sænke tempoet og få ekstra hvile. Hvis du arbejder, kan du få behov for at være sygemeldt helt eller delvist, i de måneder du får behandling.

Kvalme og opkastning: I forbindelse med behandlingen kan du få kvalme og kaste op. Disse gener kan forebygges med kvalmestillende medicin, som du vil få udleveret.

Blodmangel, infektion og blødning: Kemoterapi nedsætter knoglemarvens produktion af blodceller. Det kan give blodmangel, infektion og blødning. Blodmangel kan vise sig ved træthed, svimmelhed, åndenød og hjertebanken. Infektioner kan vise sig ved feber. Hvis du får en temperatur, der er højere end 38,5, skal du kontakte afdelingen, da det kan være nødvendigt at behandle en evt. infektion. Blødning kan vise sig ved for eksempel næseblod, blødning fra tandkødet, blå mærker og små røde pletter i huden.

Diarré: Du får information om, hvordan du skal forholde dig i tilfælde af diarré. Du vil desuden

få udleveret kapsler, der kan stoppe eventuel diarré.
Hårtab: Du vil ikke tabe al håret af behandlingen, men muligvis fælde mere end normalt.
Samliv og seksualitet: Din lyst til seksuelt samvær kan blive påvirket. Dette kan skyldes, at du er i en både fysisk og psykisk belastet situation. Der udskilles kun ubetydelige mængder kemoterapi via skedens slimhinder og gennem sæd. Du vil derfor kunne have samleje uden at skade din partner. Kemoterapi kan dog skade et foster, så du eller din partner skal anvende et svangerskabs- forebyggende middel som spiral eller p-piller under behandlingen og indtil 3 måneder efter behandlingens ophør.
Til mænd: Kemoterapi kan nedsætte kvaliteten af sæd. Det betyder at evnen til at få børn mindskes eller ophører helt. Din partner skal derfor bruge spiral eller p-piller under behandlingen og indtil 3 måneder efter behandlingens ophør
Til kvinder: Kemoterapi kan påvirke æggestokkene, i nogle tilfælde forbigående og i andre tilfælde vedvarende. Dette kan betyde, at menstruationen bliver uregelmæssig eller ophører, hvilket vil påvirke evnen til at få børn. Vedvarende ophør af menstruationen medfører, at du går i overgangsalderen tidligere end ellers**.** Hvis du har spørgsmål om din evne til at få børn, er du altid velkommen til at kontakte personalet for at få yderligere information.
Specielt ved Gemcitabin: Influenzalignende symptomer med feber, kulderystelser og muskelømhed kan forekomme i lettere grad efter behandling med Gemcitabin. De varer sjældent over 2 døgn og kan lindres med Panodil.
Specielt ved Capecitabin: Capecitabin-tabletterne kan give kramper i blodkarrene i hjertet og andre påvirkninger af hjertet. Dette kan opleves som brystsmerter, rytmeforstyrrelser og åndenød. Får du symptomer under behandlingen, er det vigtigt, at du informerer lægen. Du får lavet et hjertekardiogram, inden du begynder at få kemoterapi. Huden, især på dine hænder og fødder, kan blive tør, rød, revne og skalle af. Dette kan forebygges med en creme, som vil blive udleveret i ambulatoriet. Generne bliver sjældent udtalte, men kan føre til, at man må udsætte behandlingen eller nedsætte dosis.

Det kan ikke udelukkes, at der kan forekomme andre bivirkninger. Hvis vi opdager bivirkninger, som vi ikke allerede har beskrevet, vil du naturligvis blive orienteret med det samme. Herefter kan du tage stilling til, om du ønsker at fortsætte i forsøget.

T-ChOS™: Nedenstående hændelser, der er set i tidligere forsøg med T-ChOS™, er ikke vurderet at have relation til behandlingen med T-ChOS.

Milde	Appetitløshed, kvalme, øget sexlyst, åndenød, halsbrand, tidlig mæthed
Moderate	Prikkende og stikkende fornemmelse i knæregionen, øget appetit, mundtørhed, øget tørst
Alvorlige	Øgede knæsmerter, udslet på mave og brystregion, hudkløe, svimmelhed, rystelser i kroppen

Nytte ved forsøget

- Hvis behandlingen med Gemcitabin og Capecitabin i kombination med T-ChOS™ viser sig at

være mere effektiv end behandling med Gemcitabin og Capecitabin, kan det i fremtiden medføre en bedre behandling af patienter, der har/har haft kræft i bugspytkirtlen.

- Hvis projektet viser, at T-ChOS™ kan reducere vækst af kræftceller, vil det direkte nytte de patienter, der i projektet har fået denne behandling. Desværre ved vi ikke på forhånd, om du personligt vil få fordel af at deltage i forsøget.
- Projektet vil give ny værdifuld videnskabelig information om sygdomsprocesser ved kræft i bugspytkirtlen, og det kan blive til gavn for fremtidige patienter med kræft i bugspytkirtlen.

Forsøgspersoners ansvar

Du skal:

- overholde dine forsøgsaftaler. Hvis du ikke kan overholde en aftale, skal du kontakte forsøgslægen eller forsøgspersonalet for at lave en ny aftale, så snart du ved, at du ikke kan overholde den pågældende aftale.
- informere forsøgslægen eller forsøgspersonalet om eventuelle symptomer, lægekonsultationer eller indlæggelser, som du har haft eller om eventuelle ændringer i din øvrige medicin.
- informere forsøgslægen eller forsøgspersonalet, hvis du har mistanke om, at du eller din partner er gravid.
- undlade at deltage i andre forskningsprojekt, mens du deltager i dette forsøg - medmindre du har fået forsøgslægens godkendelse. Dette er for for at sikre at projektet overholder de videnskabelige regler og for at beskytte dig mod mulige skader, som kunne opstå i forbindelse med f.eks. ekstra blodprøvetagning, eventuelle vekselvirkninger mellem forskellige slags forsøgslægemidler eller lignende risici.

Graviditet

Kvinder i den fødedygtige alder skal anvende en effektiv præventionsmetode for at undgå at udsætte et foster for ukendte risici. For at sikre dette, skal der foretages en graviditetstest inden første behandling. Mænd og kvinder i den fødedygtige alder skal bruge en sikker præventionsmetode under forsøget og i 3 måneder efter sidste behandling. Følgende svangerskabsforebyggende midler anses som sikker prævention i forbindelse med dette lægemiddelforsøg: spiral eller hormonel antikonception (p-piller, implantat, transdermal depotplastre, vaginalring eller depotinjektion). Man kan også vælge at praktisere seksuel afholdenhed i hele forsøgsperioden og 3 måneder efter sidste forsøgsbehandling. Der er ikke krav om brug af prævention, hvis du eller din partner enten ved operation eller på grund af andre omstændigheder er sterile. Hvis du er i tvivl om, at du anvender sikker prævention, kan du altid rådføre dig med den læge, der er ansvarlig for din behandling.
Hvis din partner bliver gravid, mens du deltager i dette forsøg, eller inden for 90 dage efter at behandlingen er ophørt, skal du meddele det til forsøgslægen eller forsøgspersonalet. Da risikoen for partneren og barnet er ukendt, vil din partner blive bedt om at underskrive en samtykkeerklæring, som tillader medicinsk opfølgning vedrørende udfaldet af graviditeten.

Forsikring for forsøgsdeltagere

I Danmark er alle deltagere i forsøg med lægemidler/kosttilskud omfattet af Lov om klage og erstatningsadgang inden for sundhedsvæsenet. Skulle der mod forventning opstå en helbredsskade som følge af din deltagelse i forsøget, er du omfattet af Lov om klage og erstatningsadgang inden for sundhedsvæsenet og Bekendtgørelse om dækningsområde for lov om patientforsikring. Anmodning om erstatning for skader skal du sende til Patientforsikringen.
Du mister ingen juridiske rettigheder ved at underskrive samtykke- og fuldmagtserklæringen.

Hvem får at vide, at jeg deltager i forsøget?

Det er tilladt repræsentanter fra GCP-enheden ved Københavns Universitet, Lægemiddelstyrelsen, Fødevarestyrelsen, Videnskabsetisk Komité eller tilsvarende udenlandsk myndighed at få adgang til oplysninger i din journal. Alle personer, der er involveret i forsøget, har tavshedspligt og vil behandle personlige data strengt fortroligt. Der vil på intet tidspunkt blive udleveret oplysninger, som kan henføres til dig personligt. Hvis du vælger at trække dit informerede samtykke tilbage, vil ingen nye data blive indsamlet og registreret. Imidlertid tillader lovgivningen, at data, der er indsamlet, inden du trækker dit samtykke tilbage, stadig indgår i forsøgets datamateriale. Oplysningerne vil blive registret og analyseret i en computer. Opbevaring af data vil følge gældende regler, og lov om behandling af personoplysninger vil blive overholdt. De indsamlede data vil blive opbevaret i 20 år efter forsøgets afslutning og vil blive anvendt i en videnskabelig opgørelse. Herefter vil de blive destrueret.

Økonomi

Hospitalet vil modtage 14.000 kr. for hver forsøgsperson, der deltager i forsøget. Disse penge modtages fra det islandske firma GENIS, der har udviklet T-ChOS^{TM} fra rejeskaller. Beløbet indsættes på hospitalets forskningskonto, der administreres af hospitalet og er under offentlig kontrol. Disse penge skal anvendes til de ekstra udgifter, der er i relation til projektet. Herudover har personalet, som behandler dig (den forsøgsansvarlige læge, andre læger eller sygeplejersker) og det øvrige forsøgspersonale ingen økonomiske fordele af, at du deltager i forsøget.
Du får ikke betaling for at deltage i dette forskningsforsøg.
Der vil desuden blive søgt om økonomisk støtte fra offentlige såvel som private fonde til gennemførelsen af projektet. Ingen fonde eller sponsorer vil få nogen rolle i eller indflydelse på forsøget.
De videnskabsetiske komiteer og deltagerne vil blive orienteret, hvis der opnås fondsstøtte.

Yderligere information/Kontaktpersoner

Forsøgslægen eller forsøgspersonalet vil besvare eventuelle spørgsmål, du måtte have.

Forsøgsansvarlig læge:

Afdelingslæge, Inna Chen

Onkologisk Afdeling, Herlev Hospital

[redacted]

Mail: [redacted]

Forsøgsansvarlig projektsygeplejerske:
Onkologisk Afdeling, Herlev Hospital
Tlf.:
Mail:

27.3. APPENDIX: INFORMED CONSENT (IN DANISH), VERSION 1.0, 2015-10-21

Informeret samtykke til deltagelse i et sundhedsvidenskabeligt forskningsforsøg

Titel:

En randomiseret, dobbelt blindt placebo-kontrolleret fase II undersøgelse af T-ChOS™ eller placebo i kombination med standardbehandling med Gemcitabin og Capecitabin, som adjuverende (forebyggende) behandling til patienter, der er opereret for kræft i bugspytkirtlen

Erklæring fra forsøgsperson:

Jeg har fået skriftlig og mundtlig information, og jeg ved nok om formål, metode, fordele og ulemper til at sige ja til at deltage. Jeg ved, det er frivilligt at deltage, og at jeg altid kan trække mit samtykke tilbage uden at miste mine nuværende eller fremtidige rettigheder til behandling.

Hvis der kommer nye væsentlige helbredsoplysninger frem om dig i forskningsprojektet, vil du blive informeret.
Vil du **frabede** dig information om nye væsentlige helbredsoplysninger, som kommer frem i forskningsprojektet, bedes du markere det her: _________ (sæt x).

Forsøgspersonens navn: ____

Dato:___________________________________ Underskrift:______________________________

Ønsker du at blive informeret om forskningsprojektets resultat og eventuelle konsekvenser for dig?:

Ja: ______ (sæt x) Nej: ______ (sæt x)

Erklæring fra den der afgiver information:
Jeg erklærer, at forsøgspersonen har modtaget mundtlig og skriftlig information om forsøget. Efter min overbevisning er der givet tilstrækkelig information til, at der kan træffes beslutning om deltagelse i forsøget.

Navnet på den, der har givet informationen: ____

Dato og Underskrift: ____

Fuldmagt til deltagelse i et sundhedsvidenskabeligt forskningsforsøg

Titel:

En randomiseret, dobbelt blindt placebo-kontrolleret fase II undersøgelse af T-ChOS™ eller placebo i kombination med standardbehandling med Gemcitabin og Capecitabin, som adjuverende (forebyggende) behandling til patienter, der er opereret for kræft i bugspytkirtlen

Fuldmagt

For at der kan føres tilsyn under og efter forsøget, giver jeg hermed fuldmagt til, at relevante danske som udenlandske myndigheder har adgang til min patientjournal i 20 år. Fuldmagten kan til enhver tid tilbagekaldes. Alle oplysninger behandles strengt fortroligt.

Jeg har modtaget en kopi af den underskrevne og daterede deltagerinformation og fuldmagtserklæring.

Forsøgspersonens underskrift: ______________________________Dato: ____________

____

Forsøgspersonens navn med blokbogstaver

27.4. APPENDIX: FIRST CONTACT AND INFORMED CONSENT GUIDANCE (IN DANISH), VERSION 1.0, 2015-08-20

Første kontakt til forsøgspersonen og retningslinjer for afgivelse af mundtlig information og indhentning af samtykke

Første kontakt

Patienten vil blive set i Onkologisk Afdelings ambulatorium.

Informationssamtale

1. **Inden informationssamtalen**
   - skal der træffes aftale om tid og sted for samtalen
   - skal der oplyses om, at der er tale om en forespørgsel om deltagelse i et videnskabeligt forsøg
   - skal der oplyses om retten til betænkningstid efter information og muligheden for at medbringe bisidder til samtalen

2. **Informationssamtalen**
   - skal være nøje planlagt
   - skalforegå i uforstyrrede rammer
   - deltageren skal gives tilstrækkelig tid til at læse den skriftlige information, lytte til den mundtlige information og stille spørgsmål (den skriftlige information udleveres efter at den mundtlige information er givet)
   - investigator skal oplyse deltageren om retten til at frasige sig viden om egne helbredsforhold
   - informationen gives af den studieansvarlige læge eller af den dertil bemyndigede person (subinvestigator = læge) med tilknytning til forsøget

3. **Indhentelse af samtykke**
   - patientens samtykke til forsøgsdeltagelse afgives snarest efter informationssamtalen dog under hensyntagen til fornøden betænkningstid, som er mindst et døgn

27.5. NOTIFICATION OF SERIOUS ADVERSE EVENT (SAE)

For the Sponsor	Received (date/signature):	SAE no:
**CHIPAC**	**Serious Adverse Event**	
**Patient number** ☐☐☐☐☐	**Patient age:**<br>**Patient sex: male** ☐<br>**female** ☐	
Investigator name	Type of report: Initial ☐Follow up☐	
Treatment: gemcitabine + capecitabin + T-ChOS™ / placebo	Date of onset of SAE ☐☐☐☐☐☐☐☐	
Date of last dose of T-ChOS™ /placebo ☐☐☐☐☐☐☐☐		
Date of last dose of gemcitabine ☐☐☐☐☐☐☐☐		
Date of last dose of capecitabin ☐☐☐☐☐☐☐☐		

Reason for considering the event serious: Fatal ☐ Life-threatening☐

Hospitalization/prolonged hospital stay☐ Disabling/incapacitating☐

Associated with congenital abnormality☐ Other event☐ specify: _______________

Diagnosis/description of event:

……

……

Type of toxicity: ……………......................... CTCAE Grade1☐ 2☐ 3☐ 4☐ 5☐

Relation to T-ChOS™ /placebo:	Not related☐	Unlikely☐	Probable☐	Certain☐
Relation to gemcitabine:	Not related☐	Unlikely☐	Probable☐	Certain☐
Relation to capecitabine:	Not related☐	Unlikely☐	Probable☐	Certain☐

Action taken regarding T-ChOS™ /placebo:	None☐	Discontinued☐	Dose reduction☐	Delayed☐	Reduction of infusion rate☐
Action taken regarding gemcitabine:	None☐	Discontinued☐	Dose reduction☐	Delayed☐	Reduction of infusion rate☐
Action taken regarding capecitabine:	None☐	Discontinued☐	Dose reduction☐	Delayed☐	Reduction of infusion rate☐

Names and doses of drug(s) given to treat the SAE:

……...

……...

Other actions to treat the event: No☐ Yes☐

Describe:

Outcome Recovered☐ Recovered with sequelae☐ Ongoing☐ Unknown☐

Dead☐ Date of outcome/death: ☐☐☐☐☐☐☐☐

Signature of person completing the form Date ☐☐☐☐☐☐☐☐

Investigator's signature Date ☐☐☐☐☐☐☐☐

All dates as ddmmyyyy CTO, Oncology Department, Herlev University Hospital CHIPAC SAE-form, version 1.0, 2015-08-20

27.6. APPENDIX: EORTC QUALITY OF LIFE QUESTIONNAIRE C30 (QLQ-C30) VERSION 3.0

EORTC QLQ-C30 (version 3.0)

Vi er interesserede i at vide noget om dig og dit helbred. Vær venlig at besvare alle spørgsmålene selv ved at sætte en ring omkring det svar (tal), som passer bedst på dig. Der er ingen "rigtige" eller "forkerte" svar. De oplysninger, som du giver os, vil forblive strengt fortrolige.

Skriv venligst dine forbogstaver her: |_|_|_|_|
Din fødselsdato (dag, måned, år): |_|_|_|_|_|_|_|_|
Dato for udfyldelse af dette skema (dag, måned, år): 31 |_|_|_|_|_|_|_|_|

	Slet ikke	Lidt	En del	Meget
1. Har du nogen vanskeligheder ved at udføre anstrengende aktiviteter, som f.eks. at bære en tung indkøbstaske eller en kuffert?	1	2	3	4
2. Har du nogen vanskeligheder ved at gå en lang tur?	1	2	3	4
3. Har du nogen vanskeligheder ved at gå en kort tur udendørs?	1	2	3	4
4. Er du nødt til at ligge i sengen eller at sidde i en stol om dagen?	1	2	3	4
5. Har du brug for hjælp til at spise, tage tøj på, vaske dig eller gå på toilettet?	1	2	3	4

**I den forløbne uge:**	Slet ikke	Lidt	En del	Meget
6. Var du begrænset i udførelsen af enten dit arbejde eller andre daglige aktiviteter?	1	2	3	4
7. Var du begrænset i at dyrke dine hobbyer eller andre fritidsaktiviteter?	1	2	3	4
8. Havde du åndenød?	1	2	3	4
9. Har du haft smerter?	1	2	3	4
10. Havde du brug for at hvile dig?	1	2	3	4
11. Har du haft besvær med at sove?	1	2	3	4
12. Har du følt dig svag?	1	2	3	4
13. Har du savnet appetit?	1	2	3	4
14. Har du haft kvalme?	1	2	3	4
15. Har du kastet op?	1	2	3	4

Vær venlig at fortsætte på næste side

I den forløbne uge:	Slet ikke	Lidt	En del	Meget
16. Har du haft forstoppelse?	1	2	3	4
17. Har du haft diarré (tynd mave)?	1	2	3	4
18. Var du træt?	1	2	3	4
19. Vanskeliggjorde smerter dine daglige gøremål?	1	2	3	4
20. Har du haft svært ved at koncentrere dig om ting som f.eks. at læse avis eller se fjernsyn?	1	2	3	4
21. Følte du dig anspændt?	1	2	3	4
22. Var du bekymret?	1	2	3	4
23. Følte du dig irritabel?	1	2	3	4
24. Følte du dig deprimeret?	1	2	3	4
25. Har du haft svært ved at huske?	1	2	3	4
26. Har din fysiske tilstand eller medicinsk behandling vanskeliggjort dit familieliv?	1	2	3	4
27. Har din fysiske tilstand eller medicinsk behandling vanskeliggjort din omgang med andre mennesker?	1	2	3	4
28. Har din fysiske tilstand eller medicinsk behandling medført økonomiske vanskeligheder for dig?	1	2	3	4

Ved de næste 2 spørgsmål bedes du sætte en ring omkring det tal mellem 1 og 7, som passer bedst på dig

29. Hvordan vil du vurdere dit samlede helbred i den forløbne uge?

1	2	3	4	5	6	7
Meget dårligt						Særdeles godt

30. Hvordan vil du vurdere din samlede livskvalitet i den forløbne uge?

1	2	3	4	5	6	7
Meget dårlig						Særdeles god

© Copyright 1995 EORTC Study Group on Quality of Life. All rights reserved. Version 3.0
